# Timing of an antioxidant–acidifier feed additive modulates growth performance and cecal microbiota in broiler chickens during enrofloxacin exposure

**DOI:** 10.1016/j.psj.2026.107639

**Published:** 2026-08-17

**Authors:** Ádám Kerek, Máté Hetyésy, Gergely Álmos Tornyos, Eszter Kaszab, Enikő Fehér, Ákos Jerzsele, Tamás Tóth, Eszter Zsédely, Hedvig Fébel

**Affiliations:** a Department of Pharmacology and Toxicology, University of Veterinary Medicine Budapest, István utca 2, H-1078, Budapest, Hungary; b National Laboratory of Infectious Animal Diseases, Antimicrobial Resistance, Veterinary Public Health and Food Chain Safety, University of Veterinary Medicine Budapest, István utca 2, H-1078, Budapest, Hungary; c Department of Bioinformatics, One Health Institute, Faculty of Health Sciences, University of Debrecen, Nagyerdei krt. 98, H-4032, Debrecen, Hungary; d Department of Microbiology and Infectious Diseases, University of Veterinary Medicine, István u 2, H-1078, Budapest, Hungary; e National Laboratory of Virology, Szentágothai Research Centre, University of Pécs, H-7624, Pécs, Hungary; f Agricultural and Food Research Center, Széchenyi István University, Egyetem Square 1, H-9026, Győr, Hungary; g Department of Animal Science, Széchenyi István University, Vár square 2, H-9200, Mosonmagyaróvár, Hungary; h Department of Obstetrics and Food Animal Medicine Clinic, University of Veterinary Medicine Budapest, István utca 2, H-1078, Budapest, Hungary

**Keywords:** Broiler chicken, Enrofloxacin, Cecal microbiota, Fumaric acid, Antioxidant–acidifier feed additive

## Abstract

Nutritional interventions targeting oxidative stress and gut ecology may help sustain broiler performance during antimicrobial perturbation. This study evaluated the timing-dependent effects of an antioxidant–acidifier feed additive, alone or combined with enrofloxacin, on growth performance and cecal microbiota in broilers. A total of 1,200 Ross 308 male broilers were assigned to six treatments (4 pens/treatment; 50 birds/pen) and reared to 42 d: control, enrofloxacin (d15–19), additive for 4 weeks (A4; d15–42), A4+enrofloxacin, additive for 6 weeks (A6; d1–42), and A6+enrofloxacin. The additive was included at 4% of the diet, providing fumaric acid, heat-stable vitamin C, and all-rac-α-tocopheryl acetate. Performance traits were recorded by phase and overall. Cecal contents were collected from 2 birds/pen on d14, d20, and d42 and analyzed by shotgun metagenomic sequencing; microbiota analyses were conducted on pen-level aggregated profiles. Growth performance showed modest, timing-dependent numerical differences: A4 had numerically higher finisher and overall average daily gain, whereas A4+enrofloxacin was associated with lower overall gain than enrofloxacin alone. Cecal community structure was driven primarily by sampling time point. No detectable enrofloxacin-associated separation was observed at d20, whereas a trend toward separation was evident at d42. By d42, only limited genus-level differences were detected, with the strongest signal observed for *Phocaeicola* in the targeted differential abundance analysis. Overall, additive timing influenced growth responses and cecal microbiota trajectories during enrofloxacin exposure, supporting the importance of supplementation timing when nutritional strategies are used to improve robustness under antibiotic perturbation.

## Introduction

Antimicrobial use remains essential for safeguarding broiler health and welfare, yet it can also select for resistant bacteria and resistance determinants of One Health relevance. In this context, One Health refers to the interconnected health of animals, humans, and the shared environment, particularly with respect to antimicrobial use and resistance dissemination across ecological compartments (Roth et al., 2019; Tang et al., 2023). Poultry production occupies a central position in this landscape: it is globally expanding, biologically efficient, and tightly integrated into international food chains, because high-throughput production systems can couple dense animal populations and recurrent infectious pressure with antimicrobial interventions (Colomer-Lluch et al., 2011; Roth et al., 2019; Muteeb et al., 2023). Antimicrobials remain indispensable for safeguarding welfare and productivity under disease challenge; nevertheless, repeated or poorly targeted use can contribute to the selection and dissemination of resistant bacteria and resistance determinants across ecological compartments (Roth et al., 2019; Mulchandani et al., 2023; Tang et al., 2023; Lőrincz et al., 2025; Farkas et al., 2025). Consequently, contemporary poultry science increasingly seeks interventions that (i) preserve flock performance and intestinal functionality under stressors and (ii) reduce reliance on antimicrobials by supporting resilience and recovery of the host–microbiota system.

Beyond resistance selection, antibiotic administration is a strong ecological perturbation for the gastrointestinal tract, where the microbiota constitutes a highly dynamic community with key roles in nutrient utilization, immune maturation, colonization resistance, and maintenance of mucosal homeostasis (Diaz Carrasco et al., 2019; Mandal et al., 2020; Varga-Balogh and Olasz, 2025). In broilers, early-life microbial assembly is particularly sensitive to environmental and nutritional inputs; disruptions during this window may shift developmental trajectories of the gut ecosystem with downstream consequences for growth and health (Mandal et al., 2020; Somogyi and Farkas, 2025). Experimental and field observations indicate that antimicrobial exposure can reshape community structure, alter microbial diversity, and influence the abundance of antibiotic resistance genes in the gut, with partial restoration occurring after withdrawal but with time- and context-dependent dynamics (Temmerman et al., 2022; Ma et al., 2023).

This is especially relevant for fluoroquinolones such as enrofloxacin, which are used in poultry medicine in specific clinical contexts but may influence the intestinal resistome reservoir given their relevance in human medicine and their broad activity spectrum (Subhadra et al., 2019; Peña et al., 2025).

In parallel, modern broiler genotypes exhibit exceptionally rapid growth and high metabolic turnover, which can increase susceptibility to oxidative and inflammatory stress, particularly under additional environmental or physiological challenges (Rigoulet et al., 2020; Masenga et al., 2023; Yang et al., 2024). Oxidative stress is not merely a systemic redox phenomenon: it intersects with gut physiology (barrier integrity, mucosal immune tone, epithelial renewal) and can modulate host–microbe interactions at multiple levels (Mandal et al., 2020; Masenga et al., 2023). Moreover, oxidative pressure can influence bacterial stress responses, including membrane adaptation and protective pathways that may indirectly affect antimicrobial tolerance phenotypes (Duda-Madej et al., 2025; Gulcin, 2025; Somogyi and Farkas, 2025). This multidimensional coupling — antibiotic perturbation, oxidative challenge, and microbiota instability — supports a mechanistic rationale for nutritional strategies that aim to (i) buffer oxidative damage, (ii) stabilize intestinal conditions, and (iii) facilitate post-challenge recovery of a functional microbiota.

Feed-based interventions with antioxidants and acidifying components are among the most practical approaches for large-scale poultry systems because they can be implemented uniformly and continuously without handling stress. Antioxidant strategies commonly include vitamins and synthetic phenolic antioxidants that protect lipids and cellular structures against peroxidation and may support immune competence under stress (Vraka et al., 2017; Rostagno, 2020; Khalifa et al., 2021; Hayanti et al., 2022; Hetényi et al., 2024; Zhou et al., 2024). Vitamin C (ascorbic acid) is often discussed in poultry nutrition due to its roles in redox homeostasis and stress adaptation, including potential benefits under heat and other stressors (Shojadoost et al., 2021; Hieu et al., 2022). Vitamin E (α-tocopherol), as a lipid-phase antioxidant, is a central component of protection against membrane lipid peroxidation and has been linked to improved oxidative status, immune-related outcomes and growth of poultry (Vraka et al., 2017; Khalifa et al., 2021; Hayanti et al., 2022). Organic acids — used as acidifiers and functional additives — can modulate gastrointestinal pH, influence nutrient digestibility, and exert antimicrobial effects in a pH-dependent manner, thereby contributing to a more favorable gut environment (Skinner et al., 1991; He et al., 2020). Fumaric acid, in particular, has been used in poultry feeds as an acidifier; its potential benefits include support of performance and mitigation of intestinal challenges through physicochemical and microbial mechanisms (Skinner et al., 1991; He et al., 2020).

Despite extensive use of antioxidants and organic acids, two translational gaps remain prominent. First, supplementation timing is likely critical: early-life administration may shape microbial assembly and intestinal maturation differently than later-life supplementation, yet comparative timing data for complex additives are limited and often not evaluated alongside a defined antimicrobial perturbation (Mandal et al., 2020). Second, studies frequently rely on performance outcomes alone, but the intestinal ecosystem — particularly the cecal microbiota — may carry important explanatory signals for both growth responses and post-antibiotic recovery (Diaz Carrasco et al., 2019; Mandal et al., 2020; Sebők et al., 2024). Recent work applying high-throughput microbiome and resistome profiling under fluoroquinolone exposure demonstrates that antibiotic dose and treatment windows can produce distinct microbiota/resistome signatures, with partial normalization following withdrawal (Temmerman et al., 2022; Ma et al., 2023). In parallel, shotgun metagenomics is increasingly used to move beyond broad taxonomic patterns toward functional inference (e.g., gene repertoires related to resistance and microbial metabolism), reinforcing the cecum as a key reservoir for genetic richness relevant to health and production (Peña et al., 2025). To our knowledge, direct comparisons of early-life versus later supplementation with an antioxidant–acidifier feed additive under a defined, short enrofloxacin exposure, coupled with longitudinal cecal microbiota profiling across pre- and post-treatment timepoints, remain limited.

Therefore, the objective of the present study was to evaluate whether the timing of an antioxidant–acidifier feed additive modifies growth performance and cecal microbiota trajectories in broilers under a defined enrofloxacin perturbation. We hypothesized that supplementation timing would differentially influence intestinal ecosystem development and the trajectory of post-antibiotic community recovery, with downstream consequences for flock robustness and production-related endpoints. The study was not designed to demonstrate a uniform protective effect of the additive across all endpoints, but rather to test whether additive timing modulates the biological response to antibiotic perturbation. By integrating production metrics with microbiota profiling across key developmental and post-treatment timepoints, this study aims to provide practical, mechanistically informed evidence for nutritional strategies that enhance resilience in modern broiler production and align with antimicrobial stewardship objectives.

Because the present study was designed to address production and cecal microbiota responses under field-relevant conditions, host oxidative and inflammatory biomarkers were not included; consequently, antioxidant-related mechanisms are interpreted here as biologically plausible rather than directly demonstrated. However, evidence remains limited on whether the timing of antioxidant–acidifier supplementation modulates broiler performance and cecal community trajectories under a defined fluoroquinolone perturbation. By combining a pen-replicated production trial with longitudinal shotgun metagenomics, this study provides timing-resolved, practice-relevant data to inform nutrition strategies that support robustness during antimicrobial exposure.

## Materials and methods

### Ethical approval

The study was approved by the competent Hungarian authority (Permit No. GY/20/02058-6/2024). The chickens were handled according to the ethical standards and guidelines of European Union regulations (Directive 2010/63/EU, 2010). All procedures were performed to minimize discomfort and ensure the well-being of the broilers used in this study.

### Experimental design, animals and housing

A total of 1,200 one-day-old Ross 308 male broiler chicks were used. The trial was conducted from hatch to day 42 at the poultry facility of Széchenyi István University (Mosonmagyaróvár, Hungary). Birds were reared on deep litter in pens of 50 birds per pen. Twenty-four pens were used, corresponding to four replicate pens per treatment group. A schematic overview of the experimental treatments, additive timing, and pen-level sampling scheme is provided in Supplementary Table S1. Six experimental groups were established: (1) Control (C; no feed additive and no enrofloxacin), (2) enrofloxacin only (EF), (3) feed additive for 4 weeks (A4; additive from d15 to 42), (4) additive for 4 weeks plus enrofloxacin (A4+EF), (5) feed additive for 6 weeks (A6; additive from d1 to 42), and (6) additive for 6 weeks plus enrofloxacin (A6+EF). Enrofloxacin was administered via drinking water using Baytril 100 mg/mL oral solution (A.U.V.), purchased from Tolnagro Kft. (Szekszárd, Hungary), at 10 mg/kg body weight for five consecutive days between day 15 and day 19 of age. Environmental conditions (temperature, relative humidity, lighting program, and ventilation) were set according to Ross 308 management recommendations. Feed and water were provided *ad libitum*.

### Nutrition, feed composition and analytical determination

Birds received three diets according to production phase: starter (d1 to 10), grower (d11 to 24), and finisher (d25 to 42). The composition, calculated nutrient content, and energy value of the diets are provided in Table 1, Table 2. The tested feed additive was included at 4% (w/w) in the relevant phases. In A4 and A4+EF, the additive was included from d15 (grower) through d42 (finisher); in A6 and A6+EF, it was included from d1 through d42 (all phases). The additive contained fumaric acid, heat-stable vitamin C, and vitamin E; inclusion levels in the complete feeds were 20 g/kg fumaric acid, 0.2 g/kg heat-stable vitamin C, and 115 mg/kg all-rac-α-tocopheryl acetate. The fumaric acid was used at the maximum inclusion level permitted under current regulations. Considering the effective vitamin content of the commercial products used for C and E vitamin supplementation in the feed additive, the final vitamin content provided by supplementation in the diets was 70 mg L-ascorbate-2-monophosphate/kg diet and 38.5 mg d-α-tocopherol/kg diet.

**Table 1:** Composition of diets (g/kg, air dry basis) during the three phases of the study.

Ingredient	Control Starter	Control Grower	Control Finisher	Additive Starter	Additive Grower	Additive Finisher
Corn	637.3	668.3	703.4	572.5	605.1	650.9
Soybean meal, extracted	230.0	192.0	146.0	245.1	205.0	150.0
Corn gluten meal	65.0	70.0	75.0	65.0	70.0	75.0
Monocalcium phosphate	17.4	15.5	14.4	17.5	15.7	14.3
Limestone (feed grade)	11.3	10.0	9.1	11.1	9.7	9.2
Vegetable oil	3.4	12.0	20.0	14.6	23.0	28.0
Antioxidant-acidifier feed additive^a^	0.0	0.0	0.0	40.0	40.0	40.0
L-Lysine HCl	7.3	6.7	6.9	7.0	6.4	6.9
Butylated hydroxytoluene	5.0	5.0	5.0	5.0	5.0	5.0
Sodium bicarbonate	4.3	3.9	3.9	4.3	3.9	3.9
Sodium chloride	1.0	1.2	1.2	1.0	1.2	1.2
L-Arginine	3.9	3.5	3.8	3.6	3.3	3.8
L-Threonine	2.8	2.1	2.1	2.4	2.1	2.1
DL-Methionine	4.3	3.6	3.5	4.3	3.7	3.6
L-Valine	1.9	1.5	1.5	1.8	1.4	1.6
L-Isoleucine	1.8	1.6	1.8	1.6	1.4	1.8
L-Tryptophan	0.4	0.4	0.4	0.3	0.3	0.5
Choline chloride	0.8	0.8	0.6	0.8	0.8	0.6
Trace mineral premix^b^	1.0	1.0	1.0	1.0	1.0	1.0
Vitamin premix^c^	0.5	0.5	0.5	0.5	0.5	0.5
MAXIBAN G160^d^	0.6	0.5	0.0	0.6	0.5	0.0

a Feed additive provided the following per kg of complete feed: 20 g fumaric acid (Yantai Hengyuan Bioengineering Co., Shandong, China); 0.2 g ROVIMIX STAY-C 35 (DSM, Heerlen, The Netherlands); 0.115 g Vitamin E 50% WS (ORFFA Additives Ltd., Breda, The Netherlands).

b Trace mineral premix provided per kg of complete feed: 120.6 mg Mn (MnO and MnSO₄·H₂O); 109.9 mg Zn (ZnO and ZnSO₄·H₂O); 48.6 mg Fe (FeSO₄·H₂O); 16.0 mg Cu (CuSO₄·5H₂O); 1.2 mg I (Ca(IO₃)₂); 0.4 mg Se (Na₂SeO₃ and selenomethionine).

c Vitamin premix provided per kg of complete feed as follows: Starter diet: vitamin A, 12,000 IU (retinyl acetate); cholecalciferol, 5,000 IU; vitamin E, 80 mg (dl-α-tocopheryl acetate); riboflavin, 10.6 mg; thiamine, 3.6 mg; pantothenic acid, 16.55 mg; niacin, 59.8 mg; folic acid, 2.1 mg; biotin, 0.18 mg; pyridoxine, 5.76 mg; cyanocobalamin, 0.038 mg; menadione, 3.6 mg. Grower diet: vitamin A, 10,000 IU (retinyl acetate); cholecalciferol, 4,500 IU; vitamin E, 65 mg (dl-α-tocopheryl acetate); riboflavin, 10.6 mg; thiamine, 3.6 mg; pantothenic acid, 16.55 mg; niacin, 59.8 mg; folic acid, 2.1 mg; biotin, 0.18 mg; pyridoxine, 5.76 mg; cyanocobalamin, 0.038 mg; menadione, 3.6 mg. Finisher diet: vitamin A, 9,000 IU (retinyl acetate); cholecalciferol, 4,000 IU; vitamin E, 55 mg (dl-α-tocopheryl acetate); riboflavin, 10.6 mg; thiamine, 3.6 mg; pantothenic acid, 13.98 mg; niacin, 55.8 mg; folic acid, 1.7 mg; biotin, 0.18 mg; pyridoxine, 5.76 mg; cyanocobalamin, 0.024 mg; menadione, 3.6 mg.

d MAXIBAN G160 (Elanco Products Co., Hook, Hampshire, UK).

**Table 2:** Calculated nutrient composition (%) and metabolizable energy content of the diets.

Nutrient	Control Starter	Control Grower	Control Finisher	Additive Starter	Additive Grower	Additive Finisher
AMEn (kcal/kg)*	3000	3100	3200	3000	3100	3200
Crude protein (%)	21.50	20.00	18.50	21.50	20.00	18.50
Ether extract (%)	3.81	4.68	5.49	4.74	5.59	6.03
Crude fiber (%)	2.63	2.46	2.24	2.61	2.42	2.17
Ash (%)	6.04	5.53	5.10	6.04	5.52	5.06
Lysine (%)	1.44	1.29	1.19	1.44	1.29	1.19
Methionine (%)	0.76	0.68	0.65	0.76	0.68	0.79
Methionine + cystine (%)	1.08	0.99	0.94	1.08	0.99	0.94
Threonine (%)	1.00	0.88	0.81	0.97	0.88	0.81
Tryptophan (%)	0.23	0.21	0.19	0.23	0.21	0.19

⁎ AMEn: apparent metabolizable energy corrected to zero nitrogen retention and equivalent MJ/kg values are 12.55, 12.97, and 13.39.

Feed samples were collected from each diet for analysis of dry matter, crude protein, crude fiber, ether extract, and ash. All chemical analyses were performed following the AOAC methods (2012). Vitamin E concentration of experimental diets fed in our study was measured by high-performance liquid chromatography (HPLC) (Shimadzu, Japan), according to the method published by Analytical Methods Committee (1991). The content of L-ascorbate-2-monophosphate in feeds was quantified by reverse-phase HPLC with UV detection at 254 nm (AFIA, 1999). Analyzed nutrient contents and vitamin levels of diets are presented in Table 3.

**Table 3:** Analyzed nutrient composition (g/kg, as-fed basis) and vitamin levels (mg/kg, as-fed basis) of the diets.

Item	Control Starter	Control Grower	Control Finisher	Additive Starter	Additive Grower	Additive Finisher
Dry matter (g/kg)	936.6	938.9	931.2	930.1	928.6	931.8
Crude protein (g/kg)	217.6	202.8	190.4	216.4	204.7	189.6
Ether extract (g/kg)	39.7	43.9	53.7	46.1	54.2	59.4
Crude fiber (g/kg)	30.4	28.7	23.4	29.2	27.1	22.9
Ash (g/kg)	60.2	57.0	52.0	62.0	56.8	51.3
L-ascorbate-2-monophosphate (mg/kg)	< 5	< 5	< 5	72	70	71
d-α-tocopherol (mg/kg)	53	51	46	85	80	78

### Growth performance measurements, cecal sampling and storage

Individual body weight was recorded on days 1, 10, 24, and 42 (aligned with diet changes). Feed residues were weighed at the end of each feeding phase, and new feed was provided for the subsequent phase. Average daily gain (ADG) was calculated per bird for each phase, and feed intake and feed conversion ratio (FCR) were evaluated per pen. For all performance analyses, the pen was considered the experimental unit (*n* = 4 pens/treatment). Feed intake and FCR were adjusted for mortality and these corrected values were used for statistical analyses. For mortality analyses, only unscheduled deaths were counted; birds removed at the predefined cecal sampling time points (d14, d20, and d42) were excluded from mortality records and were not treated as mortality events.

Cecal sampling was performed at three time points selected relative to the enrofloxacin window: pre-treatment (d14), immediately post-treatment (d20), and end of production (d42). From each pen, two birds were randomly selected at each sampling point (total *n* = 8 birds/treatment/time point). Birds were euthanized by percussive blow to the head in accordance with animal welfare requirements. The abdominal cavity was opened, ceca were excised, and cecal contents were collected aseptically into sterile tubes containing 1.5 mL phosphate-buffered saline. Phosphate-buffered saline was used solely to facilitate sample transfer and transport. Samples were transported on dry ice and stored at −80°C until further processing.

### Metagenomic DNA extraction and Oxford Nanopore sequencing

Total metagenomic DNA was extracted using the ZymoBIOMICS DNA Miniprep Kit (Zymo Research, USA) according to the manufacturer’s protocol. DNA quantity was assessed using a Qubit fluorometer (Thermo Fisher Scientific, Waltham, MA, USA). Shotgun metagenomic sequencing was performed on an Oxford Nanopore MinION Mk1B device (Oxford Nanopore Technologies, Oxford, UK) using the Native Barcoding Kit 24 V14 SQK-NBD114.24 and FLO-MIN114 (R10.4.1) flow cells, following ONT specifications. Basecalling (with super accuracy basecalling mode) and demultiplexing of the long-read raw sequences were performed with Dorado v7.6.8 (Oxford Nanopore Technologies, 2025) as implemented in MinKNOW v 24.11.10.

Long reads were preprocessed using NanoLyse v1.2.1 and NanoFilt, v2.8.0 and quality metrics were inspected with NanoPlot v1.43.0 (De Coster et al., 2018). We performed *de novo* assembly using metaFlye v2.9.5-b1801 (Kolmogorov et al., 2020). Taxonomic classification was conducted with Kraken2 (v2.1.3) using an NCBI RefSeq Complete Genome bacterial database, and species-level abundance was estimated with Bracken; only taxa with >0.1% relative abundance were retained. For downstream ecological analyses, genus-level count tables were aggregated to pen level by summing counts across the two birds sampled per pen at each time point, yielding one community profile per pen per time point. For diversity analyses, data were normalized in QIIME 2 (version: 2025.7), beta-diversity was computed using Bray–Curtis dissimilarity, and alpha-diversity was calculated using Shannon and Simpson indices. Ordination was performed using principal coordinates analysis (PCoA). Read processing and taxonomic profiling were conducted using the ONT wf-metagenomics workflow (v2.13.0) with the corresponding workflow reference database build. Quality filtering followed the workflow defaults, and taxa below 0.1% relative abundance were excluded from downstream ecological analyses. Assembly summary and reference-based metrics (e.g., N50/L50, total length, genome fraction, misassemblies, mismatch/indel rates) were obtained using MetaQUAST. Complete taxonomic table (genus level) is provided as an online-only Supplementary Excel file.

### Statistical analysis

Performance outcomes (BW, ADG, ADFI, and FCR) were analyzed at the pen level (experimental unit; *n* = 4 pens/treatment) using a 3 × 2 factorial model with additive (organic acid + antioxidant) timing (none, A4, A6), enrofloxacin exposure (No/Yes), and their interaction as fixed effects. When appropriate, planned contrasts were extracted from the fitted models and are reported as mean differences (Δ) with 95% confidence intervals. Model assumptions were evaluated by visual inspection of residual-versus-fitted plots and normal Q–Q plots, together with the Shapiro–Wilk test for residual normality and Levene’s test for homogeneity of variances. No major deviations were detected that would warrant an alternative modelling approach. No major deviations were detected that would invalidate the applied linear models. Given the pen-level design (*n* = 4 pens per treatment), effect sizes and 95% confidence intervals are reported alongside P values to support interpretation of the magnitude and direction of treatment effects.

For microbiota analyses, two birds per pen were sampled at each time point. To avoid pseudoreplication, genus-level metagenomic counts were aggregated within pen and time point (counts summed across the two birds), yielding one community profile per pen per time point (*n* = 4 pens/treatment/time point). Aggregated counts were converted to relative abundances for community analyses. Alpha diversity (Shannon and Simpson indices) was calculated from genus-level profiles and compared using the same factorial structure within each sampling time point. Beta diversity was assessed using Bray–Curtis dissimilarity and visualized by principal coordinates analysis (PCoA); community differences were tested by PERMANOVA (adonis2, vegan) with 9,999 permutations.

For targeted differential abundance at d42, the 14 most abundant genera were analyzed on centered log-ratio (CLR)–transformed relative abundances (pseudocount = 1). A linear model including additive timing and enrofloxacin exposure was fitted, and the enrofloxacin effect (Yes − No) is reported with 95% confidence intervals. P values were adjusted using the Benjamini–Hochberg false discovery rate (FDR) within the targeted set. All analyses were performed in R (v4.1.0). Statistical significance was set at P < 0.05, whereas 0.05 ≤ P < 0.10 was interpreted conservatively as indicating a tendency.

## Results

### Growth performance

Body weight (BW) was comparable across treatments at placement (d1), indicating balanced baseline conditions (Table 4). By d42, mean BW ranged from 2406 ± 44 g (A4+EF) to 2579 ± 45 g (A4), without significant main effects or interaction in the factorial model (additive timing: P = 0.723; enrofloxacin: P = 0.290; additive timing × enrofloxacin: P = 0.178). Summary pen-level BW and ADG data are reported in Table 4, the underlying raw pen-level performance dataset is provided in Supplementary Table S3, whereas sample-level read and assembly metrics are reported in Supplementary Table S2.

**Table 4:** Body weight (BW) and average daily gain (ADG) of broilers across treatments (pen-level).

Treatment	n (pens)	BW d1	BW d10	BW d24	BW d42	ADG d1-10	ADG d11-24	ADG d25-42	ADG d1-42
		g				(g/bird/d)			
Control	4	40.9 ± 0.3	222 ± 6	983 ± 13	2480 ± 71	18.2 ± 0.6	54.0 ± 1.3	83.1 ± 3.3	58.1 ± 1.7
Enrofloxacin	4	40.9 ± 0.3	214 ± 5	1005 ± 18	2529 ± 53	17.3 ± 0.4	56.6 ± 1.2	83.8 ± 3.3	59.0 ± 1.5
A4	4	41.3 ± 0.2	223 ± 8	988 ± 16	2579 ± 45	18.2 ± 0.8	54.6 ± 1.1	88.1 ± 1.8	60.4 ± 1.1
A4+enrofloxacin	4	40.7 ± 0.2	221 ± 4	954 ± 12	2406 ± 44	17.9 ± 0.4	52.4 ± 0.8	80.6 ± 2.3	56.3 ± 1.0
A6	4	40.9 ± 0.4	196 ± 14	956 ± 28	2474 ± 64	15.4 ± 1.4	54.2 ± 1.1	83.3 ± 2.8	57.4 ± 1.7
A6+enrofloxacin	4	40.9 ± 0.2	212 ± 5	976 ± 17	2445 ± 63	17.2 ± 0.6	53.8 ± 0.9	81.6 ± 3.0	57.2 ± 1.5
**P-value (Additive timing)**		0.923	0.080	0.274	0.723	0.078	0.251	0.795	0.675
**P-value (Enrofloxacin)**		0.390	0.777	0.854	0.290	0.753	0.988	0.230	0.349
**P-value (Additive timing × Enrofloxacin)**		0.328	0.266	0.240	0.178	0.224	0.123	0.354	0.223

Values are means ± SEM.

The pen was the experimental unit (n = 4 pens per treatment; 50 birds per pen).

Body weight (BW) was recorded on d1, d10, d24 and d42. ADG was calculated for the starter (d1 to 10), grower (d11–24), finisher (d25 to 42), and overall (d1 to 42) periods.

Treatment groups: Control, Enrofloxacin (EF), 4-week antioxidant supplement (A4), A4+EF, 6-week antioxidant supplement (A6), and A6+EF.

P-values are from a two-factor model including additive timing (none, A4, A6), enrofloxacin exposure (yes/no), and their interaction.

### Average daily gain

**Fig. 1:**
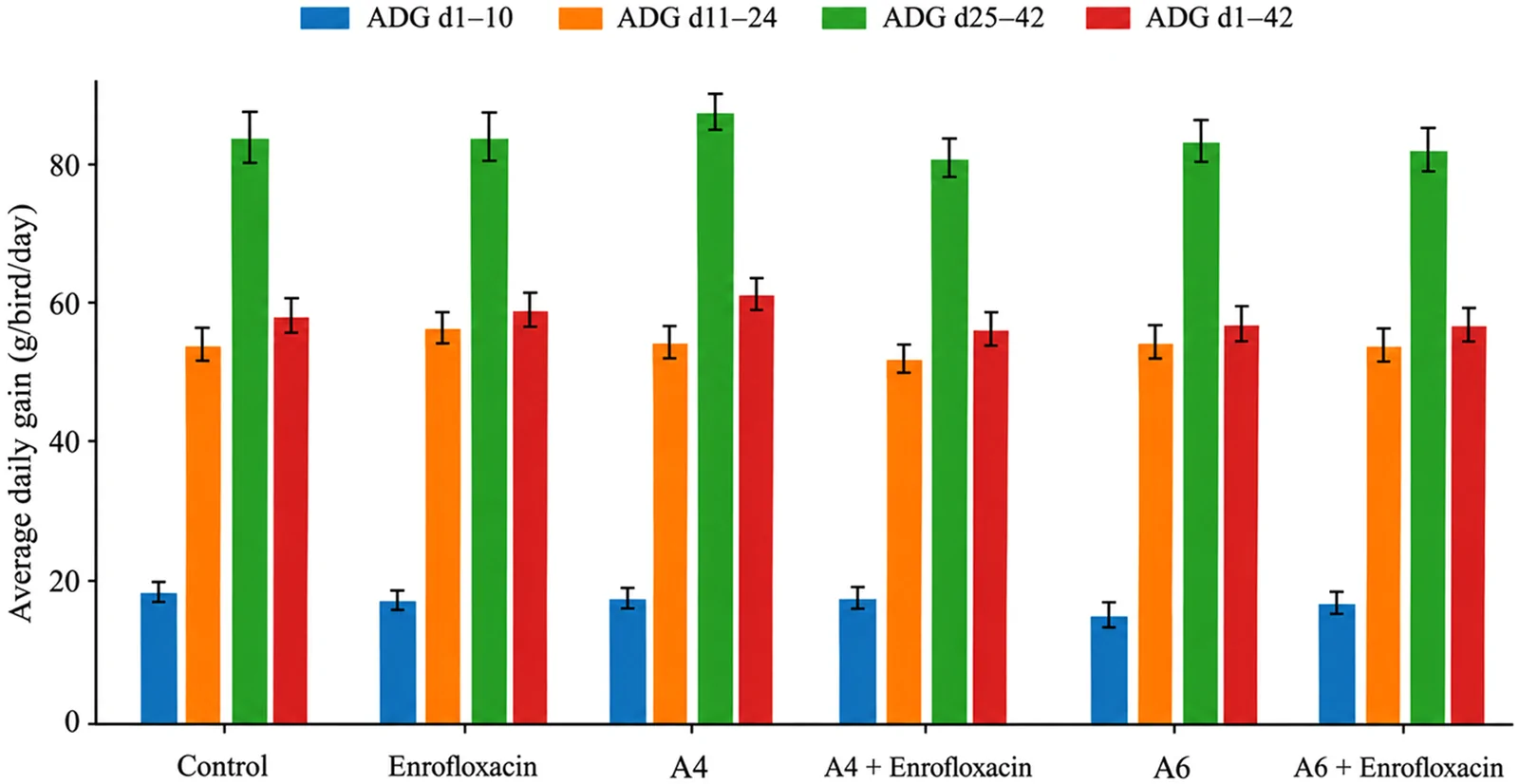
Average daily gain (ADG) by production phase across treatments (pen-level). Bars represent means ± SEM (*n* = 4 pens per treatment; pen as the experimental unit). ADG is shown for the starter (d1 to 10), grower (d11–24), finisher (d25 to 42), and overall (d1 to 42) periods. Treatment groups: Control (C), Enrofloxacin (EF), 4-week antioxidant supplement (A4), A4+EF, 6-week antioxidant supplement (A6), and A6+EF. Statistical comparisons were performed using a two-factor model including additive timing, enrofloxacin exposure, and their interaction.

Average daily gain (ADG) increased with age in all groups and reached its maximum during the finisher phase (d25 to 42), with pen-level means ranging from 80.6 to 88.1 g/bird/d (Table 4; Fig. 1). Numerically, the highest finisher ADG was observed in A4 (88.1 ± 1.8 g/bird/d) and the lowest in A4+EF (80.6 ± 2.3 g/bird/d); however, these differences were not statistically significant in the factorial model (additive timing: P = 0.795; enrofloxacin: P = 0.230; additive timing × enrofloxacin: P = 0.354).

Across the entire rearing period (d1 to 42), overall ADG ranged from 56.3 to 60.4 g/bird/d. The highest mean overall ADG was recorded in A4 (60.4 ± 1.1 g/bird/d) and the lowest in A4+EF (56.3 ± 1.0 g/bird/d), without significant main effects or interaction (additive timing: P = 0.675; enrofloxacin: P = 0.349; additive timing × enrofloxacin: P = 0.223). Phase-specific ADG results are summarized in Table 4 and visualized in Fig. 1.

### Feed intake and feed conversion

Mortality-corrected average daily feed intake (ADFI) increased with age in all groups and was highest during the finisher phase (d25 to 42), with pen-level means ranging from 135.8 to 144.4 g/bird/d (Table 5). Across the entire rearing period (d1 to 42), total ADFI ranged from 83.9 to 88.6 g/bird/d, with no significant main effects or interaction in the factorial model (additive timing: P = 0.202; enrofloxacin: P = 0.606; additive timing × enrofloxacin: P = 0.187). In planned contrasts, finisher ADFI tended to be higher in A4 than in the control (Δ = +7.2 g/bird/d, 95% CI: −0.9 to +15.2; P = 0.077), whereas total ADFI tended to be lower in A6 than in the control (Δ = −4.2 g/bird/d, 95% CI: −8.8 to +0.4; P = 0.069). In the grower phase (d11 to 24), ADFI differed by additive timing (P < 0.001), whereas enrofloxacin exposure and the interaction were not significant (enrofloxacin: P = 0.342; additive timing × enrofloxacin: P = 0.632).

**Table 5:** Mortality-corrected average daily feed intake (ADFI) and feed conversion ratio (FCR) by phase across treatments (pen-level).

Treatment	n (pens)	ADFI (g/bird/d)					FCR (kg feed/kg gain)			
		d1–10	d11–24	d25–42	d1–42	d1–10		d11–24	d25–42	d1–42
Control	4	22.3 ± 0.9	78.3 ± 1.2	137.2 ± 2.7	88.1 ± 1.3	1.249 ± 0.018		1.569 ± 0.022	1.662 ± 0.035	1.599 ± 0.022
Enrofloxacin	4	19.7 ± 0.8	78.1 ± 0.8	140.1 ± 2.8	87.6 ± 1.2	1.208 ± 0.027		1.539 ± 0.039	1.763 ± 0.094	1.640 ± 0.042
A4	4	22.8 ± 1.1	71.7 ± 1.3	144.4 ± 1.9	88.6 ± 1.2	1.318 ± 0.057		1.420 ± 0.024	1.657 ± 0.046	1.559 ± 0.021
A4+enrofloxacin	4	22.2 ± 0.5	69.3 ± 0.6	135.9 ± 2.1	84.9 ± 1.1	1.241 ± 0.027		1.436 ± 0.018	1.687 ± 0.025	1.573 ± 0.013
A6	4	17.9 ± 2.6	73.1 ± 1.9	135.8 ± 3.6	83.9 ± 2.5	1.244 ± 0.062		1.510 ± 0.071	1.663 ± 0.054	1.585 ± 0.035
A6+enrofloxacin	4	21.0 ± 0.7	72.8 ± 0.9	137.3 ± 2.8	86.1 ± 1.4	1.239 ± 0.014		1.455 ± 0.037	1.686 ± 0.030	1.578 ± 0.015
P-value (Additive timing)		0.096	<0.001	0.431	0.202	0.412		0.017	0.694	0.155
P-value (Enrofloxacin)		0.983	0.342	0.539	0.606	0.210		0.485	0.249	0.479
P-value (Additive timing × Enrofloxacin)		0.119	0.632	0.099	0.187	0.666		0.670	0.714	0.667

Values are means ± SEM.

The pen was the experimental unit (n = 4 pens per treatment; 50 birds per pen).

ADFI values are mortality-corrected and expressed as g/bird/day.

FCR values are mortality-corrected and expressed as kg feed/kg gain.

ADFI and FCR are reported for the starter (d1 to 10), grower (d11 to 24), finisher (d25 to 42), and overall (d1 to 42) periods.

P-values are from a two-factor model including additive timing (none, A4, A6), enrofloxacin exposure (yes/no), and their interaction.

Treatment groups: Control (C), Enrofloxacin (EF), 4-week antioxidant supplement (A4), A4+EF, 6-week antioxidant supplement (A6), and A6+EF.

**Fig. 2:**
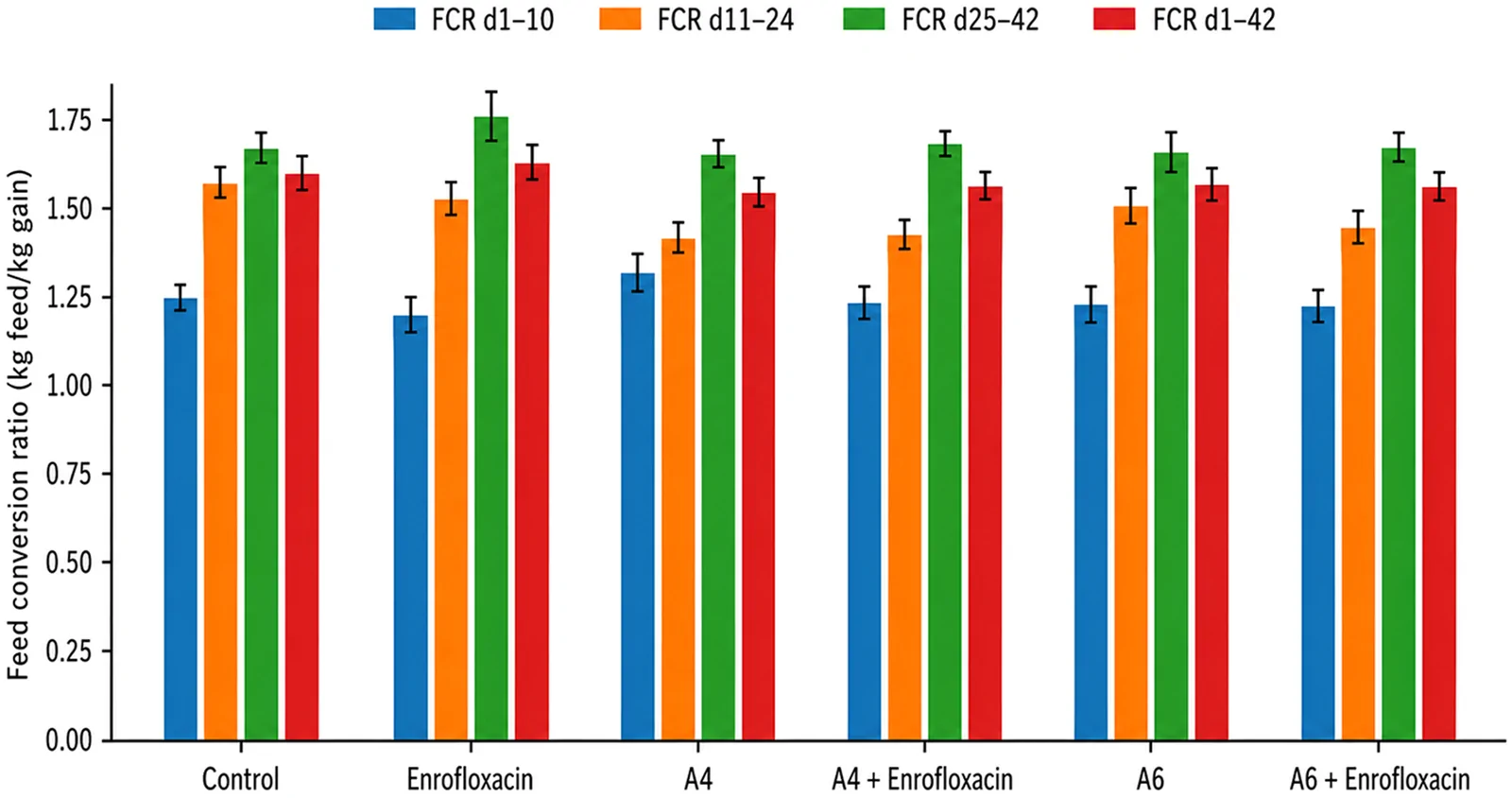
Mortality-corrected feed conversion ratio (FCR) by production phase across treatments (pen-level). Bars represent means ± SEM (*n* = 4 pens per treatment; pen as the experimental unit). FCR values are mortality-corrected and shown for the starter (d1 to 10), grower (d11 to 24), finisher (d25 to 42), and overall (d1 to 42) periods. Treatment groups: Control (C), Enrofloxacin (EF), 4-week antioxidant supplement (A4), A4+EF, 6-week antioxidant supplement (A6), and A6+EF. Statistical comparisons were performed using a two-factor model (additive timing, enrofloxacin, and their interaction).

Mortality-corrected feed conversion ratio (FCR) during the finisher phase (d25 to 42) ranged from 1.657 to 1.763 kg/kg, and total FCR (d1 to 42) ranged from 1.559 to 1.640 kg/kg (Fig. 2). Total FCR did not differ significantly among treatments (additive timing: P = 0.155; enrofloxacin: P = 0.479; additive timing × enrofloxacin: P = 0.667). A supplementation timing effect was detected for grower-phase FCR (d11 to 24; P = 0.017), with no significant effect of enrofloxacin exposure or interaction. Phase-specific ADFI and FCR results are summarized in Table 5, and FCR is visualized in Fig. 2.

### Mortality

**Fig. 3:**
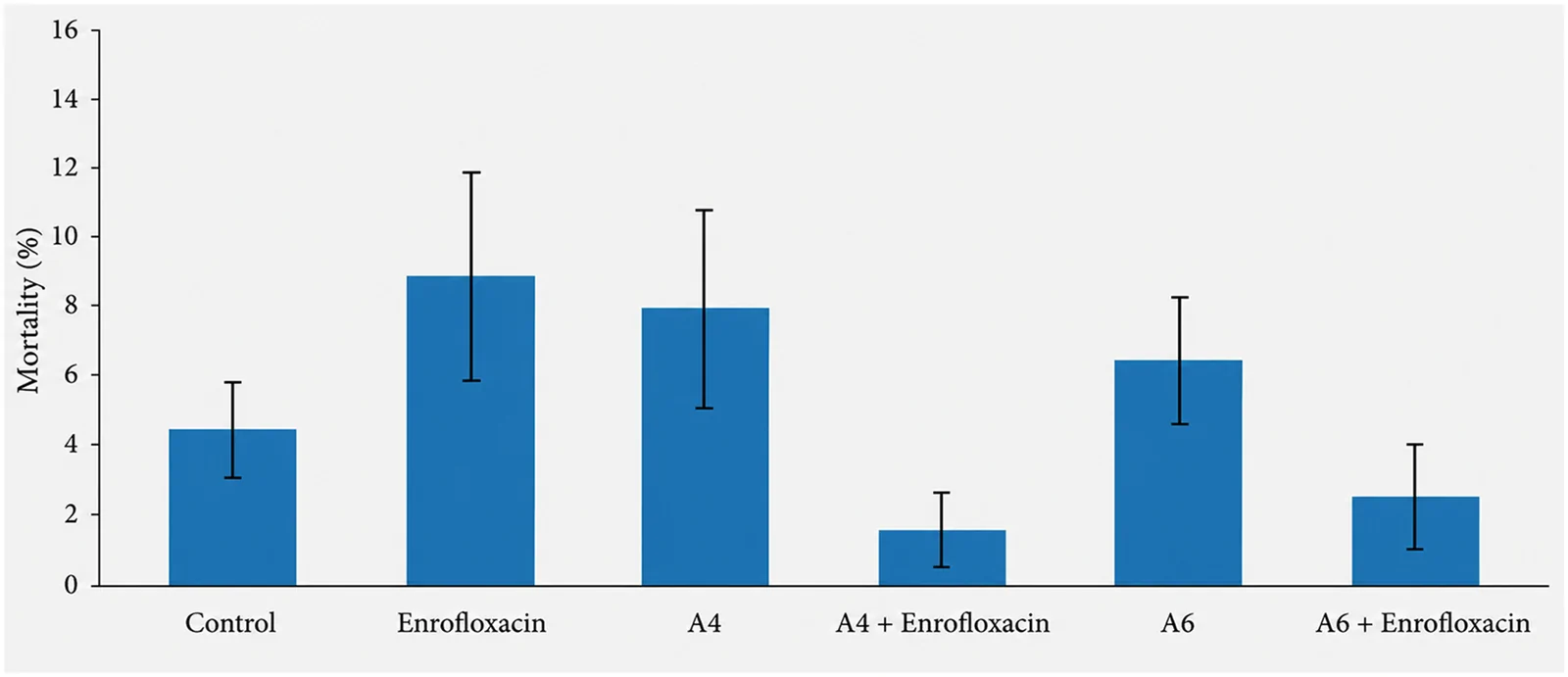
Mortality across treatments during the rearing period (d1 to 42). Bars represent pen-level means ± SEM (n = 4 pens per treatment). Mortality is expressed as percentage (%) of birds per pen and includes only unscheduled deaths; birds removed at predefined cecal sampling time points were excluded from mortality calculations. Treatment groups: Control (C), Enrofloxacin (EF), 4-week antioxidant–acidifier supplementation (A4), A4+EF, 6-week antioxidant–acidifier supplementation (A6), and A6+EF. Statistical comparisons were performed using a two-factor model including additive timing, enrofloxacin exposure, and their interaction.

Mortality differed across treatments (Table 6). Cumulative mortality was highest in EF (8.9 ± 2.9%) and in A4 (7.9 ± 2.7%), whereas the combined additive + enrofloxacin groups showed the lowest mortality (A4+EF: 1.5 ± 0.9%; A6+EF: 2.5 ± 1.3%). In the factorial model, the supplementation timing × enrofloxacin interaction was significant (P = 0.029), whereas the main effects of supplementation timing (P = 0.467) and enrofloxacin exposure (P = 0.235) were not significant (Fig. 3).

**Table 6:** Mortality during the rearing period (d1 to 42) across treatments (pen-level).

Treatment	n (pens)	Mortality (%, d1–42)	Mortality (n/pen, d1–42)
Control	4	4.4 ± 1.2	2.2 ± 0.6
Enrofloxacin	4	8.9 ± 2.9	4.5 ± 1.5
A4	4	7.9 ± 2.7	4.0 ± 1.4
A4+enrofloxacin	4	1.5 ± 0.9	0.8 ± 0.5
A6	4	6.4 ± 1.7	3.2 ± 0.9
A6+enrofloxacin	4	2.5 ± 1.3	1.2 ± 0.6
P-value (Additive timing)		0.467	
P-value (Enrofloxacin)		0.235	
P-value (Additive timing × Enrofloxacin)		0.029	

Values are means ± SEM. The pen was the experimental unit (n = 4 pens per treatment). Mortality is reported as percentage (%) and as number of birds per pen for d1 to 42. Only unscheduled deaths were included; birds removed at predefined cecal sampling time points were excluded from mortality calculations. P-values are from a two-factor model including additive timing (none, A4, A6), enrofloxacin exposure (yes/no), and their interaction. Treatment groups: Control (C), Enrofloxacin (EF), 4-week antioxidant supplement (A4), A4+EF, 6-week antioxidant supplement (A6), and A6+EF.

### Cecal microbiota profiles

Genus-level taxonomic profiles were generated from 142 individual cecal samples collected at d14 (*n* = 46), d20 (*n* = 48), and d42 (*n* = 48). For downstream community analyses, genus-level read counts from the two birds sampled per pen at each time point were aggregated to a single pen-level profile (sum of counts; at d14, two pens contributed a single sample after QC), and pen was treated as the experimental unit (*n* = 4 pens/treatment). Sequencing depth is reported at the individual-sample level for transparency (Table 7; Supplementary Figure S1), and no samples were excluded based on sequencing depth. The full genus-level relative abundance table for all samples is available in Supplementary Table S4.

**Table 7:** Sequencing depth and genus-level alpha diversity of cecal microbiota across sampling timepoints and treatment groups.

Day	Treatment	n (samples)	Classified reads (genus-level)	Richness (genera)	Shannon (genus-level)	Simpson (genus-level)
14	Control	7	146 043 ± 49 072	100 ± 18	1.95 ± 0.23	0.677 ± 0.066
14	Enrofloxacin	8	158 057 ± 38 336	89 ± 13	1.64 ± 0.06	0.610 ± 0.034
14	A4	8	204 403 ± 45 692	112 ± 18	1.76 ± 0.10	0.615 ± 0.046
14	A4+enrofloxacin	8	157 849 ± 37 436	88 ± 7	1.78 ± 0.09	0.652 ± 0.030
14	A6	8	202 540 ± 33 032	118 ± 9	1.85 ± 0.15	0.632 ± 0.050
14	A6+enrofloxacin	7	194 368 ± 40 845	122 ± 36	1.94 ± 0.19	0.687 ± 0.045
20	Control	8	239 506 ± 56 971	157 ± 33	1.99 ± 0.08	0.687 ± 0.021
20	Enrofloxacin	8	181 495 ± 41 707	177 ± 49	2.03 ± 0.17	0.654 ± 0.044
20	A4	8	174 747 ± 53 701	142 ± 44	1.88 ± 0.10	0.673 ± 0.027
20	A4+enrofloxacin	8	167 576 ± 38 431	110 ± 19	1.79 ± 0.05	0.666 ± 0.016
20	A6	8	130 821 ± 38 745	108 ± 25	2.10 ± 0.12	0.717 ± 0.039
20	A6+enrofloxacin	8	160 263 ± 36 189	104 ± 15	1.89 ± 0.09	0.691 ± 0.021
42	Control	8	205 434 ± 41 318	196 ± 41	1.73 ± 0.17	0.552 ± 0.050
42	Enrofloxacin	8	161 406 ± 35 286	131 ± 23	1.73 ± 0.12	0.604 ± 0.048
42	A4	8	219 078 ± 42 510	188 ± 36	1.60 ± 0.07	0.497 ± 0.023
42	A4+enrofloxacin	8	153 762 ± 39 879	159 ± 43	1.79 ± 0.17	0.624 ± 0.033
42	A6	8	153 486 ± 36 496	137 ± 28	1.69 ± 0.12	0.563 ± 0.046
42	A6+enrofloxacin	8	139 518 ± 44 629	130 ± 29	1.63 ± 0.14	0.539 ± 0.037

Values are means ± SEM.

Samples were collected at d14 (pre-treatment), d20 (post-treatment), and d42 (end of production).

Treatment groups: Control (C), Enrofloxacin (EF), 4-week antioxidant supplement (A4), A4+EF, 6-week antioxidant supplement (A6), and A6+EF.

Sequencing depth is reported as the number of genus-classified reads per sample (Kraken2-based classification at genus rank). Alpha diversity metrics were calculated from genus-level relative abundance profiles and include richness (number of detected genera), Shannon diversity, and Simpson index.

Sequencing depth and per-sample alpha-diversity metrics are reported descriptively at the individual-sample level; inferential diversity and compositional analyses were performed on pen-aggregated profiles with pen as the experimental unit.

Alpha diversity (Shannon index) and genus richness computed from pen-level genus profiles showed substantial overlap among treatments at each sampling time point (Supplementary Figure S2). Although diversity patterns differed across time points, within d20 and d42 no significant main effects or interactions were detected for Shannon diversity (d20: additive timing P = 0.207, enrofloxacin P = 0.362, interaction P = 0.519; d42: additive timing P = 0.878, enrofloxacin P = 0.696, interaction P = 0.609) or richness (d20: additive timing P = 0.187, enrofloxacin P = 0.845, interaction P = 0.737; d42: additive timing P = 0.479, enrofloxacin P = 0.235, interaction P = 0.690). A substantial fraction of genus-level profiles remained unassigned at the genus rank (reported as “Unknown”), accounting for approximately 50–63% of the mean relative abundance depending on time point. This unassigned fraction is expected for genus-rank classification in shotgun metagenomics due to database and rank-resolution limitations; therefore, diversity metrics were computed from the full genus-level profiles including the unassigned fraction.

**Fig. 4:**
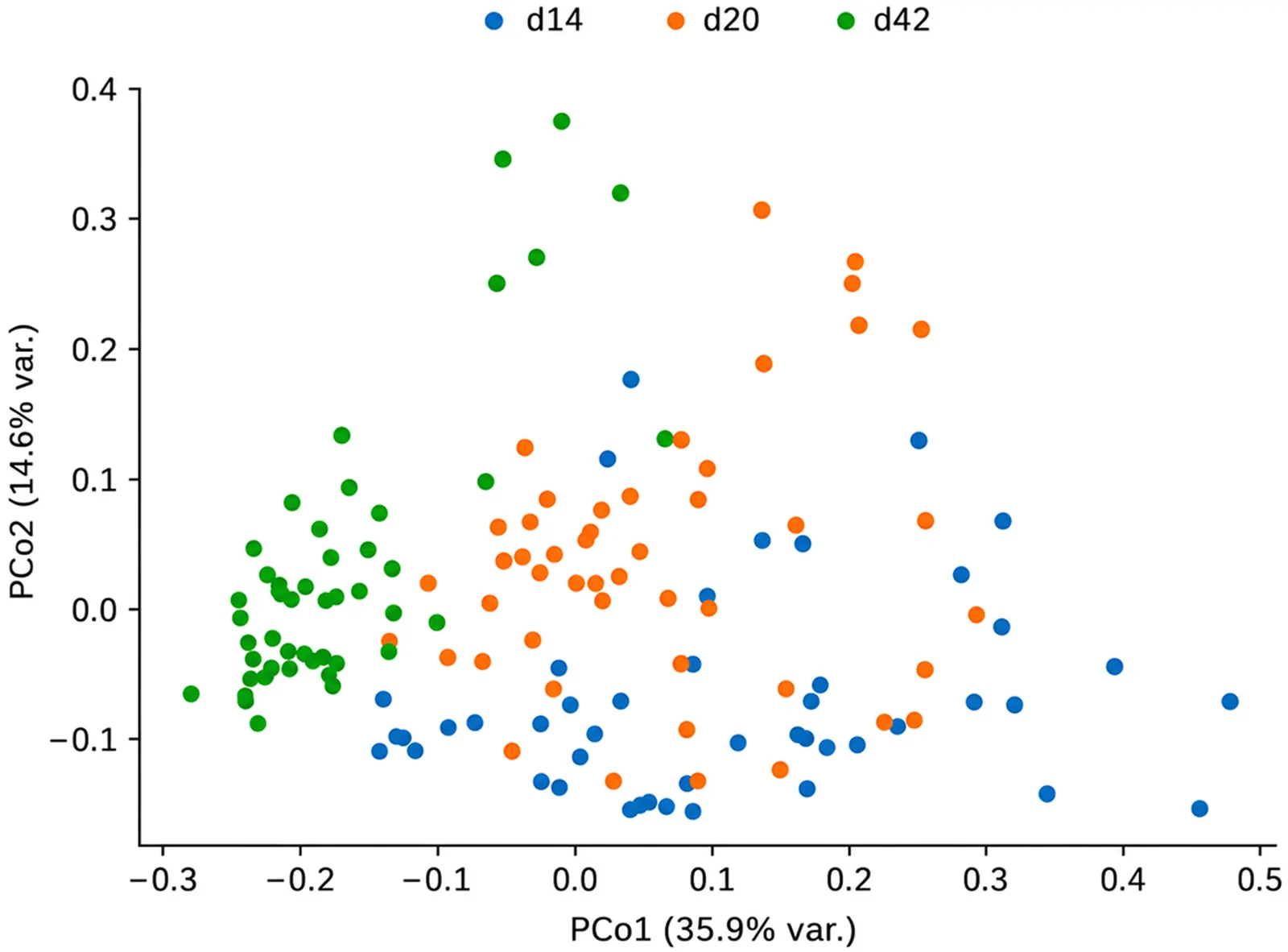
Bray–Curtis principal coordinates analysis (PCoA) of genus-level cecal community profiles across sampling time points. Genus-level read counts from the two birds sampled per pen at each time point were aggregated to a single pen-level profile (sum of counts; at d14, two pens contributed one sample after QC). Bray–Curtis dissimilarities were computed from pen-level relative abundances. Each point represents one pen. Points are colored by sampling time point (d14, d20, d42). Percent variation explained is shown on the axes.

**Fig. 5:**
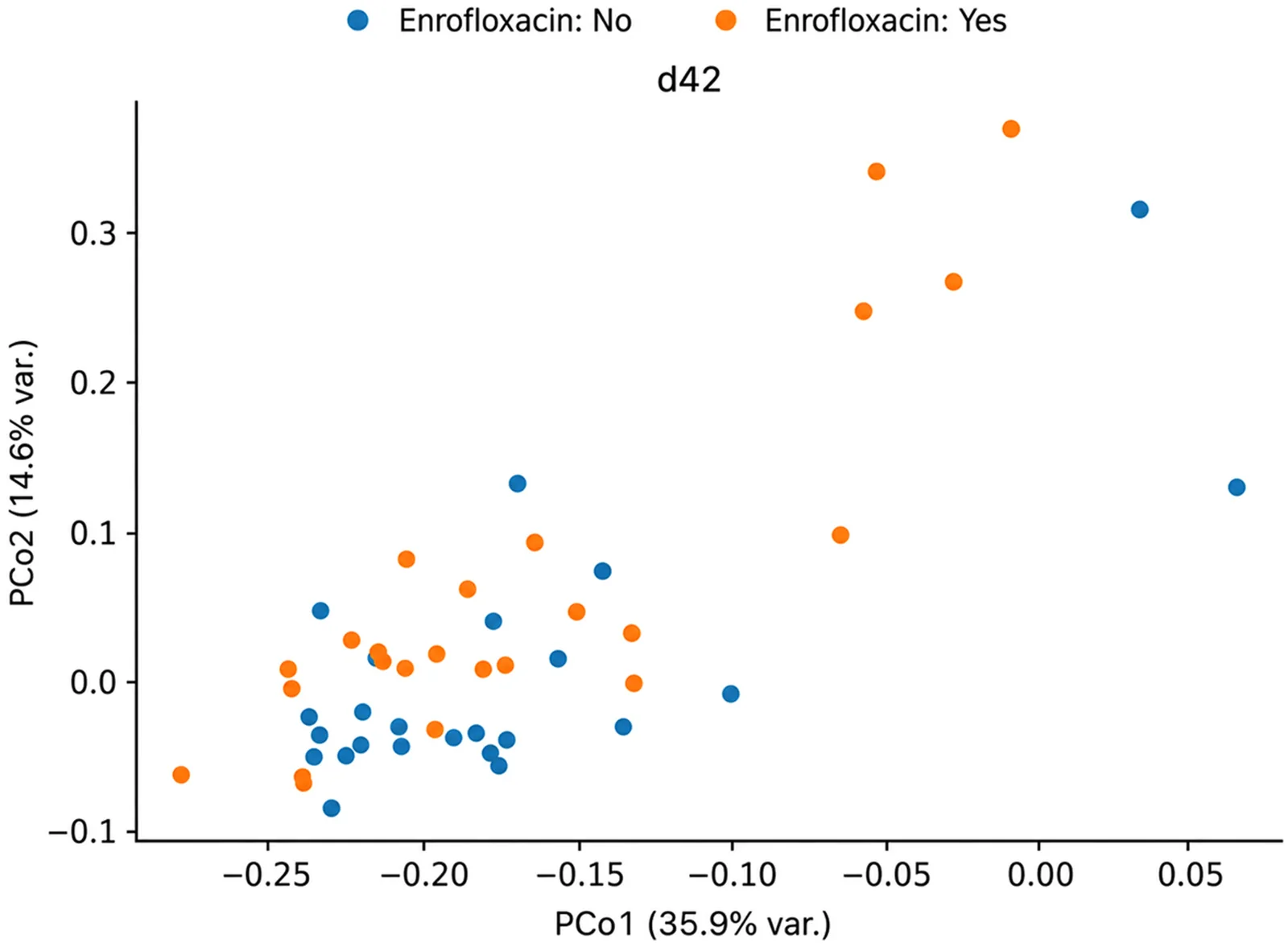
Bray–Curtis PCoA of genus-level cecal community profiles at d42 stratified by enrofloxacin exposure. Pen-level profiles were constructed by aggregating genus-level read counts from the two birds sampled per pen at d42 (sum of counts). Bray–Curtis dissimilarities were computed from pen-level relative abundances. Each point represents one pen. Points are colored by enrofloxacin exposure (Yes/No). Percent variation explained is shown on the axes.

Bray–Curtis dissimilarities were computed from genus-level relative abundances (without rarefaction), and sequencing depth distributions are shown in Supplementary Figure S1. Bray–Curtis dissimilarity revealed a strong separation by sampling timepoint (PERMANOVA: F = 34.09, R² = 0.329, P = 0.001), indicating pronounced age-associated shifts in genus-level community structure. When analyzed within timepoints, d20 showed no detectable separation by treatment group or enrofloxacin exposure (PERMANOVA: treatment R² = 0.114, P = 0.351; enrofloxacin R² = 0.007, P = 0.895; Fig. 4). At d42, treatment-group differences remained non-significant (treatment R² = 0.147, P = 0.100), but enrofloxacin exposure showed a trend toward community separation (enrofloxacin R² = 0.047, P = 0.070; Fig. 5). Pairwise PERMANOVA outputs (including FDR-adjusted P-values) and the underlying PCoA coordinates are provided in Supplementary Table S5.

### Planned contrasts and effect sizes

To complement the factorial ANOVA, pre-specified contrasts were evaluated at the pen level to quantify the magnitude and direction of the key comparisons (Table 8). These contrasts are presented primarily to describe effect direction and magnitude, not to imply statistical significance when confidence intervals overlap zero. During the finisher phase (d25 to 42), A4 showed numerically higher ADG relative to the control (Δ = +5.0 g/bird/d, 95% CI: −3.4 to +13.3; P = 0.228), whereas A6 showed no evidence of improvement versus the control (Δ = +0.2 g/bird/d, 95% CI: −8.2 to +8.5; P = 0.969). Across the full rearing period (d1–42), the direction of effects was similar (A4 vs control: Δ = +2.3 g/bird/d, 95% CI: −2.0 to +6.6; P = 0.273; A6 vs control: Δ = −0.7 g/bird/d, 95% CI: −5.0 to +3.6; P = 0.745). Within enrofloxacin-exposed birds, ADG was numerically lower in A4+EF compared with EF (finisher: Δ = −3.1 g/bird/d, 95% CI: −11.5 to +5.2; P = 0.441; overall: Δ = −2.6 g/bird/d, 95% CI: −6.9 to +1.7; P = 0.212), whereas the corresponding comparison for A6+EF was smaller and not significant. Feed intake and feed efficiency contrasts were directionally consistent with these patterns; notably, finisher ADFI tended to be higher in A4 than in the control (Δ = +7.2 g/bird/d, 95% CI: −0.9 to +15.2; P = 0.077), but overall FCR was numerically lower in A4+EF than in EF (Δ = −0.066, 95% CI: −0.146 to +0.014; P = 0.098).

**Table 8:** Planned contrasts for key performance endpoints derived from the pen-level factorial model (additive timing × enrofloxacin).

Outcome	Unit	Planned contrast	Estimate (Δ)	95% CI (low)	95% CI (high)	P value
ADG25_42	g/bird/d	A4 vs Control	4.971	−3.39	13.332	0.2276
ADG25_42	g/bird/d	A6 vs Control	0.159	−8.202	8.52	0.9686
ADG25_42	g/bird/d	A4+EF vs EF	−3.134	−11.495	5.227	0.4412
ADG25_42	g/bird/d	A6+EF vs EF	−2.162	−10.523	6.199	0.5936
ADG1_42	g/bird/d	A4 vs Control	2.316	−1.985	6.616	0.2728
ADG1_42	g/bird/d	A6 vs Control	−0.676	−4.977	3.624	0.7449
ADG1_42	g/bird/d	A4+EF vs EF	−2.647	−6.948	1.653	0.2122
ADG1_42	g/bird/d	A6+EF vs EF	−1.718	−6.019	2.582	0.4122
ADFI_finisher_gpd	g/bird/d	A4 vs Control	7.178	−0.871	15.228	0.0773
ADFI_finisher_gpd	g/bird/d	A6 vs Control	−1.401	−9.45	6.649	0.719
ADFI_finisher_gpd	g/bird/d	A4+EF vs EF	−4.23	−12.28	3.819	0.2841
ADFI_finisher_gpd	g/bird/d	A6+EF vs EF	−2.814	−10.864	5.235	0.4721
ADFI_total_gpd	g/bird/d	A4 vs Control	0.467	−4.096	5.029	0.8323
ADFI_total_gpd	g/bird/d	A6 vs Control	−4.205	−8.767	0.358	0.0687
ADFI_total_gpd	g/bird/d	A4+EF vs EF	−2.722	−7.285	1.84	0.2261
ADFI_total_gpd	g/bird/d	A6+EF vs EF	−1.5	−6.063	3.062	0.4984
FCR_finisher	ratio	A4 vs Control	−0.004	−0.161	0.152	0.9533
FCR_finisher	ratio	A6 vs Control	0.001	−0.155	0.157	0.989
FCR_finisher	ratio	A4+EF vs EF	−0.076	−0.232	0.08	0.3191
FCR_finisher	ratio	A6+EF vs EF	−0.078	−0.234	0.079	0.3103
FCR_total	ratio	A4 vs Control	−0.04	−0.12	0.04	0.3055
FCR_total	ratio	A6 vs Control	−0.013	−0.093	0.067	0.7311
FCR_total	ratio	A4+EF vs EF	−0.066	−0.146	0.014	0.0979
FCR_total	ratio	A6+EF vs EF	−0.062	−0.142	0.018	0.1197

Estimates are expressed as mean differences (Δ) with 95% confidence intervals.

Average daily gain (ADG), Daily feed intake (ADFI), Feed conversion ratio (FCR).

**Fig. 6:**
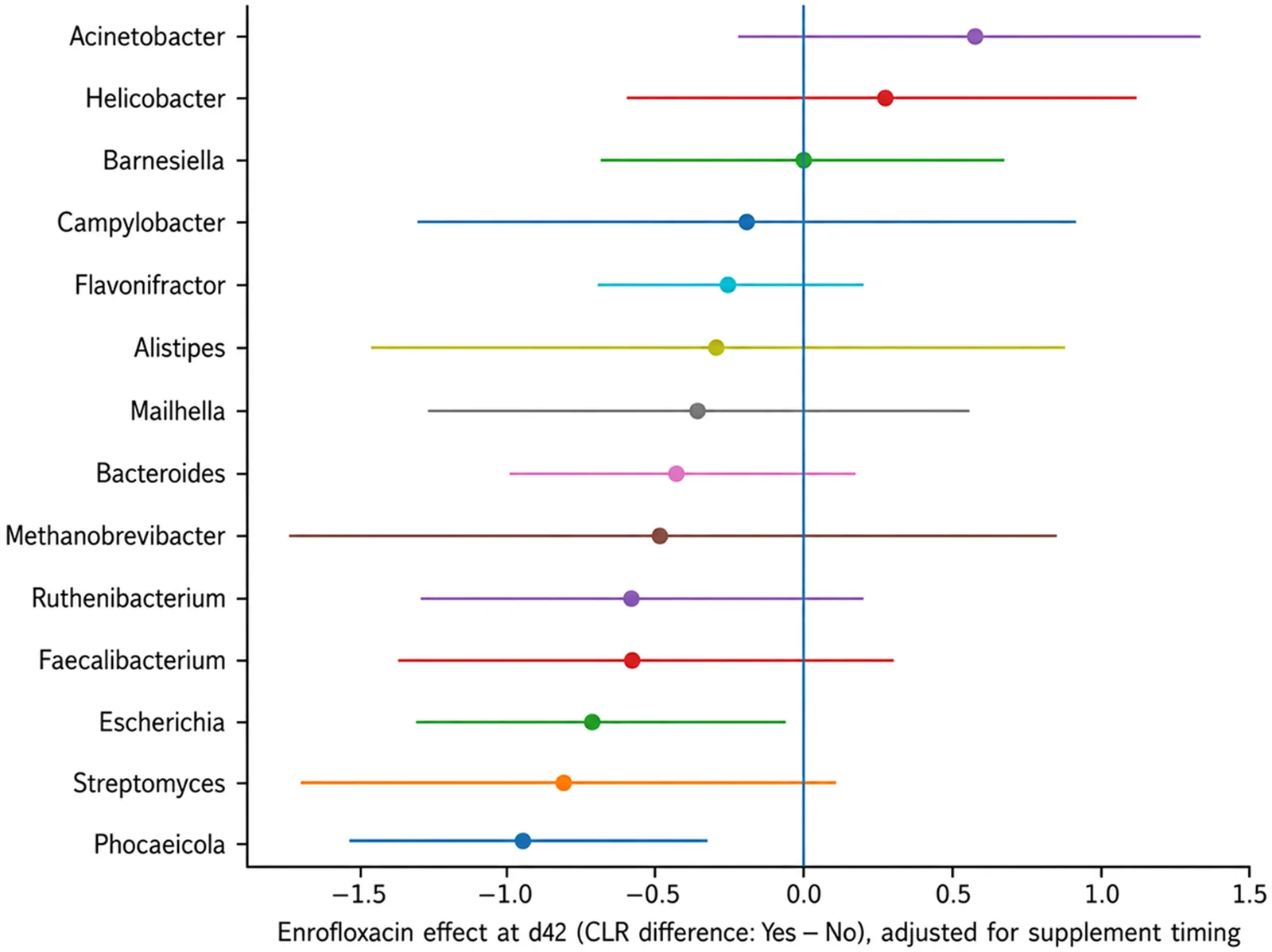
Forest plot of enrofloxacin-associated differences in CLR-transformed abundance for the 14 dominant genera at d42, estimated from OLS models adjusted for additive timing. Points indicate effect estimates (Yes − No) and horizontal lines show 95% confidence intervals.

To identify whether enrofloxacin exposure was associated with consistent shifts among the dominant cecal genera at slaughter age, a targeted differential abundance analysis was performed at d42, restricted a priori to the 14 genera with the highest mean relative abundance (Table 9; Fig. 6). Abundances were analyzed on centered log-ratio (CLR) transformed counts with supplement timing included as a covariate. Among these dominant genera, enrofloxacin exposure was associated with a lower abundance of *Phocaeicola* (EF effect: ΔCLR = −0.913, 95% CI: −1.519 to −0.306; P = 0.004; FDR q = 0.061). *Escherichia* also showed a nominal decrease with enrofloxacin exposure (ΔCLR = −0.671, 95% CI: −1.294 to −0.048; P = 0.035), but this did not remain significant after FDR adjustment across the targeted genus set (q = 0.265). No other dominant genus differed after FDR correction (Table 9).

**Table 9:** Targeted genus-level differential abundance at d42 restricted to the 14 most abundant genera (mean relative abundance).

Genus	Mean relative abundance at d42	EF effect (CLR, Yes–No)	95% CI (low)	95% CI (high)	P value	FDR q value	Mean RA (No EF)	Mean RA (EF)
Phocaeicola	0.045394	−0.912573	−1.518686	−0.30646	0.004	0.0605	0.053547	0.037241
Escherichia	0.077368	−0.671127	−1.293915	−0.048339	0.0353	0.2648	0.095177	0.059559
Streptomyces	0.011739	−0.786381	−1.681351	0.108589	0.0835	0.4176	0.013165	0.010314
Acinetobacter	0.091323	0.57097	−0.210922	1.352862	0.1482	0.4405	0.054055	0.128591
Ruthenibacterium	0.011785	−0.525173	−1.271206	0.22086	0.163	0.4405	0.013653	0.009918
Bacteroides	0.061408	−0.402401	−0.992331	0.187529	0.1762	0.4405	0.067367	0.055449
Faecalibacterium	0.01339	−0.526864	−1.364578	0.31085	0.2116	0.4535	0.015506	0.011274
Flavonifractor	0.019873	−0.236596	−0.676105	0.202913	0.2839	0.5323	0.020227	0.01952
Mailhella	0.013268	−0.343676	−1.248332	0.56098	0.448	0.6824	0.014526	0.012009
Helicobacter	0.212182	0.285159	−0.554448	1.124766	0.4973	0.6824	0.185635	0.238729
Methanobrevibacter	0.017938	−0.436874	−1.732839	0.85909	0.5005	0.6824	0.020342	0.015534
Alistipes	0.083823	−0.278849	−1.43969	0.881993	0.6307	0.7277	0.074523	0.093122
Campylobacter	0.031896	−0.185355	−1.292676	0.921967	0.7375	0.7901	0.024843	0.03895
Barnesiella	0.053017	0.008367	−0.661941	0.678675	0.98	0.98	0.049817	0.056217

Enrofloxacin effect estimates are CLR differences (Yes − No) from OLS models adjusted for additive timing; P values are nominal, and q values are Benjamini–Hochberg FDR-adjusted within the targeted set. Enrofloxacin (EF), false discovery rate (FDR), confidence interval (CI).

**Fig. 7:**
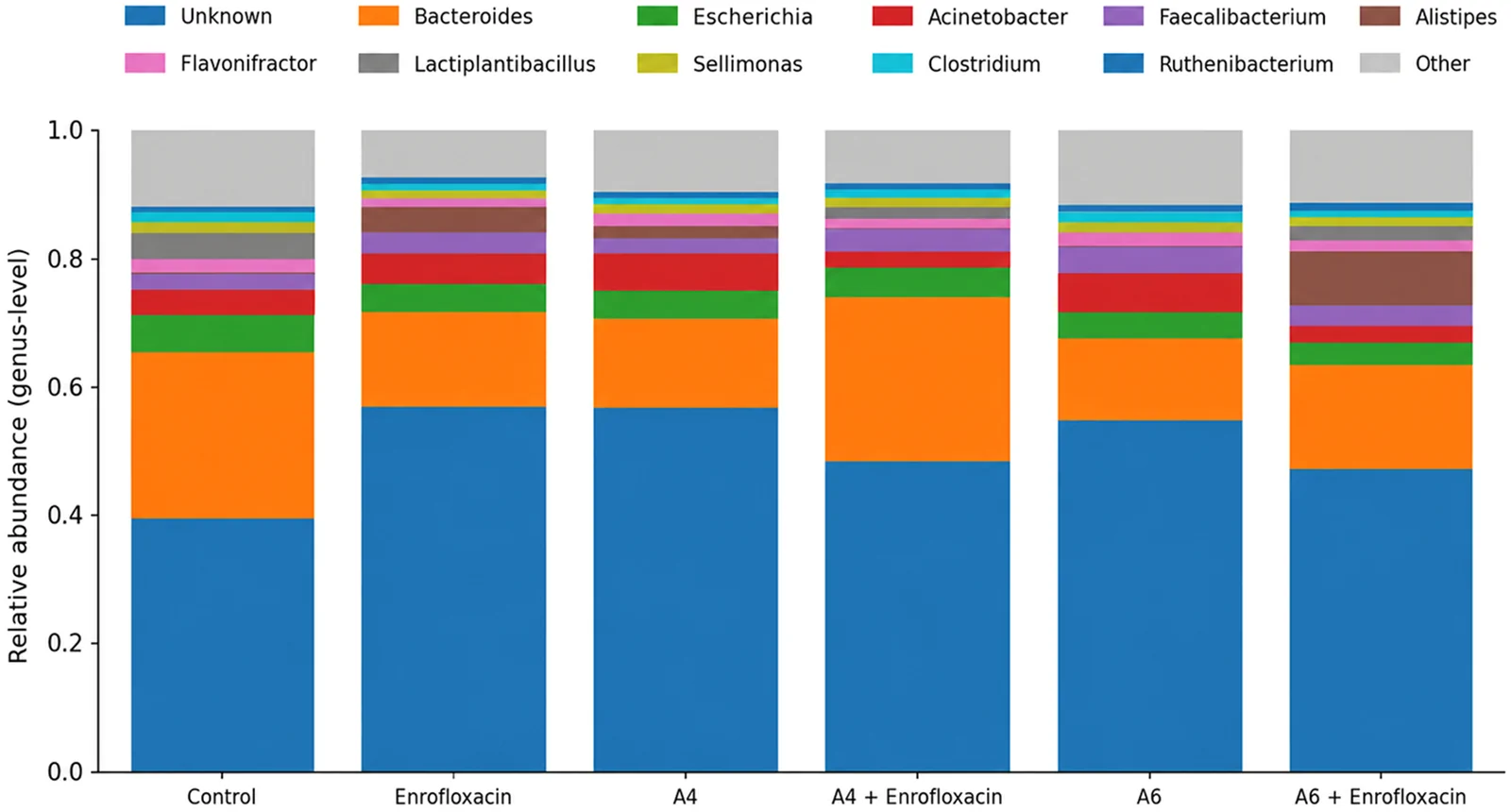
Dominant genera (mean relative abundance) across treatments at d14. Stacked bars show mean genus-level relative abundance by treatment group at d14. The top 10 genera, ranked by overall mean relative abundance at d14, are shown; remaining genera are grouped as “Other.” “Unknown” denotes reads not assigned to a genus rank.

**Fig. 8:**
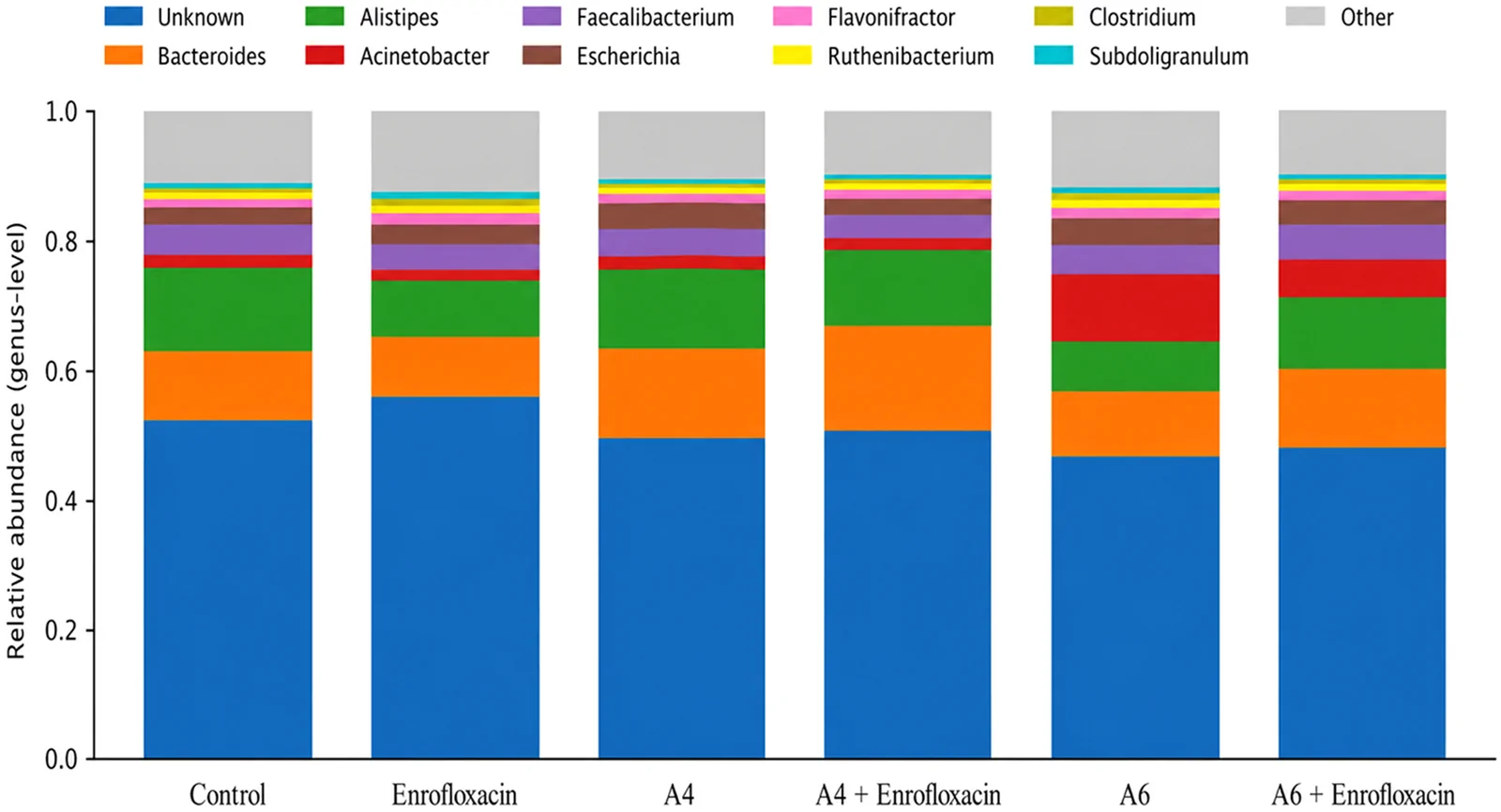
Dominant genera (mean relative abundance) across treatments at d20. Stacked bars show mean genus-level relative abundance by treatment group at d20. The top 10 genera (ranked by overall mean relative abundance at d20) are shown; remaining genera are grouped as “Other.” “Unknown” denotes reads not assigned to a genus rank.

**Fig. 9:**
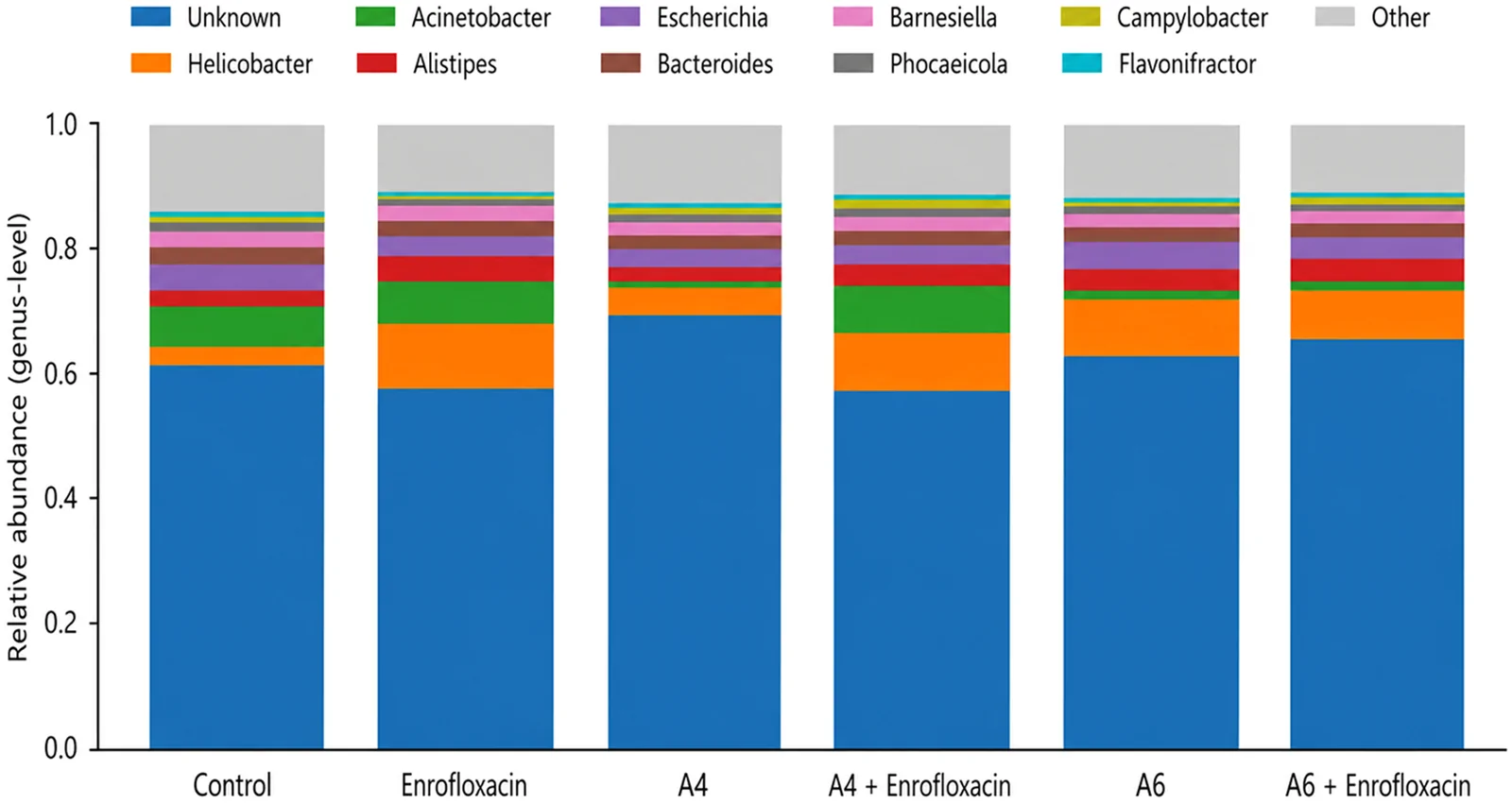
Dominant genera (mean relative abundance) across treatments at d42. Stacked bars show mean genus-level relative abundance by treatment group at d42. The top 10 genera (ranked by overall mean relative abundance at d42) are shown; remaining genera are grouped as “Other.” “Unknown” denotes reads not assigned to a genus rank.

To make the age effect explicit, dominant assigned genera were also visualized at d14. Compared with d14, the d42 cecal profiles showed reduced relative prominence of Bacteroides and Alistipes and increased contribution of Helicobacter, Acinetobacter, and Escherichia, consistent with the strong timepoint effect detected by PERMANOVA and the age-driven restructuring observed in ordination analyses (Fig. 7, Fig. 8, Fig. 9).

## Discussion

Across a full 42-d rearing period, the antioxidant–acidifier additive produced modest but structured effects on performance and gut community patterns, and these effects depended on (i) supplementation window and (ii) exposure to an antimicrobial perturbation. The central result is therefore not a single large performance shift, but a coherent pattern: (1) performance metrics showed directional differences that were generally not statistically significant under the factorial model, (2) mortality displayed an additive timing × enrofloxacin interaction, and (3) cecal community profiles remained strongly age-driven, with treatment-associated signals that were most interpretable when framed as perturbation and recovery dynamics.

In well-managed commercial production systems, growth performance can be relatively resilient to nutritional perturbations; therefore, the lack of significant main effects on ADG is not unexpected. Still, the effect-size structure is biologically informative. In the finisher phase, A4 showed the highest mean ADG, and planned contrasts suggested a positive direction versus Control (Δ ≈ +5.0 g/bird/d; CI spanning zero), but the combination of enrofloxacin with A4 was numerically lower than enrofloxacin alone (Δ ≈ −3.1 g/bird/d; CI spanning zero). Over the entire period, the same directional pattern persisted (A4 numerically highest; A4+EF numerically lowest), again with confidence intervals that overlap zero. This constellation suggests that the additive’s net performance expression may be context- and window-dependent, and that the most consequential “signal” may lie not in a mean ADG shift per se, but in whether supplementation helps birds maintain trajectory through (or after) a defined perturbation.

A second clue comes from intake-related tendencies. In planned contrasts, finisher ADFI tended to be higher in A4 than in the control (P = 0.077), whereas total ADFI tended to be lower in A6 than in the control (P = 0.069). Without overstating, this pattern raises a practical question: does a longer early-life inclusion window (A6) slightly limit intake without yielding a net performance benefit, compared with a shorter, later window (A4) that may better match the metabolic demands of birds during the final stages of the production cycle? That hypothesis remains to be tested mechanistically (e.g., palatability, acid load, or metabolic partitioning), but the present data provide a rationale for treating “additive timing” as a primary design factor rather than a secondary detail.

Although most performance differences were not statistically significant, the pattern across endpoints suggested that supplementation timing may be more influential than duration. Birds supplemented from week 3 onward (A4) consistently showed the most favorable direction of ADG and intake during the final weeks of the production period, supporting the concept that aligning nutritional support with the period of highest metabolic load can be critical for expressing small but meaningful production effects. Similar “subtle performance–stronger physiology” constellations have been reported in antioxidant-focused studies, where growth responses were limited but feed efficiency and gut barrier-related outcomes improved, together with shifts toward a more favorable microbiota profile (Xiao et al., 2024). In this context, our data are best interpreted as evidence for additive timing-sensitive trends rather than universal growth promotion.

A key translational aspect of the model is the use of fluoroquinolone perturbation. Fluoroquinolones are classified among the highest-priority medically important antimicrobials for human medicine, reinforcing the One Health relevance of strategies that can support flock robustness while limiting reliance on such drugs (GBD 2021 Antimicrobial Resistance Collaborators, 2024). In this context, the most striking outcome is the mortality pattern: enrofloxacin alone showed the highest mortality (≈8.9%), whereas both additive + enrofloxacin groups showed the lowest mortality (≈1.5–2.5%), yielding a significant additive timing × enrofloxacin interaction (P = 0.029). This does not demonstrate a causal protective mechanism, but it does indicate that the additive window meaningfully modified the system-level response to an antimicrobial perturbation in this production setting.

What could underlie such an interaction? One plausible interpretation is that enrofloxacin acts as an ecological disturbance in the gut, whereas dietary acidifying and antioxidant components may influence how the system responds to and recovers from that perturbation. The present dataset cannot disentangle host-driven from microbiota-driven contributions, yet the mortality interaction — paired with the age-structured microbiota changes — supports the idea that “performance-only” endpoints may miss the most consequential biology of perturbation–recovery.

The combination of enrofloxacin with the 4-week supplementation window (A4+EF) tended to underperform relative to A4 alone, consistent with the notion that antimicrobial perturbation can impose recovery costs that are not captured by a single endpoint. Prior work indicates that antibiotic exposure may induce persistent changes in the gut ecosystem and metabolic pathways even after withdrawal (Elokil et al., 2020), and high-dose enrofloxacin has been associated with altered intestinal physiology and community structure despite occasional performance benefits (Morales-Barrera et al., 2016). Importantly, our strongest system-level signal was observed in robustness rather than mean growth: mortality was highest under enrofloxacin alone and was markedly lower when the additive was co-administered, particularly in the A4+EF and A6+EF groups, consistent with reports that antioxidant-oriented interventions can reduce losses under stress conditions (Nath et al., 2023), but antibiotic-driven destabilization of the intestinal ecosystem has been linked to increased vulnerability in broilers (Le Roy et al., 2019).

Cecal community composition followed a clear age-associated pattern, consistent with field evidence showing that broiler cecal microbiota develop along conserved trajectories, but still reflecting between-flock and management effects (Kers et al., 2022). Against this dominant temporal signal, treatment effects are best interpreted as modulations of a moving target rather than static shifts. In other words, the meaningful question is not “does treatment change the microbiota?”, but “does treatment change how the community moves from one developmental state to the next — especially across a disturbance window?”

To keep inference rigorous, treatment-linked taxa signals were evaluated with methods attentive to the compositional nature of sequencing-based community data (Gloor et al., 2017). At day 42, targeted differential abundance analysis identified only a limited number of taxa with robust evidence after multiple-testing correction, with the strongest adjusted signal involving Phocaeicola under enrofloxacin exposure. The constrained number of significant taxa is not a weakness; it is exactly what one expects when (i) age dominates community variance, (ii) interventions are relatively moderate, and (iii) the study is designed around a realistic production environment rather than a maximally perturbed challenge model. Practically, this supports a cautious but useful conclusion: the additive did not “rewrite” the cecal microbiota; rather, it likely influenced how the system navigated perturbation and return toward a mature configuration, with any residual signature at day 42 being comparatively subtle.

At the community level, cecal microbiota profiles were dominated by age-related succession, and treatment-associated signatures were comparatively subtle. Consistent with this, enrofloxacin exposure showed only a trend-level separation of d42 community structure in Bray–Curtis space. Therefore, rather than claiming broad “microbiome normalization,” the results support a conservative interpretation in which the additive and enrofloxacin primarily modulated perturbation–recovery trajectories and only selected dominant taxa showed consistent directional shifts. This framing avoids over-interpretation and remains biologically plausible, aligning with production-relevant endpoints.

Organic acids and antioxidant vitamins are among the most scalable, low-handling interventions in broiler systems. Meta-analytic evidence indicates that organic acids can improve performance — particularly FCR — yet effects are heterogeneous and context-dependent and typically do not match antibiotic growth promoters under challenge (Polycarpo et al., 2017). This literature fits the present pattern: the feed additive’s impact on mean growth was modest, whereas the more compelling observation was a system-level interaction under antimicrobial perturbation. One mechanistic interpretation — kept explicitly as a hypothesis — is that acidification may shift luminal conditions (and thereby microbial metabolism/competition); however, antioxidants may support epithelial and immune stability under stress, together shaping resilience during and after disturbance. The present work therefore adds a practical nuance: in multi-component feed additives, timing may be as important as ingredient identity, because it determines which developmental window the intervention actually “meets” (microbiota assembly, immune maturation, or late grow-out metabolic load).

Several limitations should be stated because they define how far interpretation can go. First, pen-level replication (*n* = 4/treatment) is appropriate for a controlled production trial but limits power for detecting small treatment effects and increases uncertainty around marginal comparisons. Accordingly, the results should be interpreted primarily on the basis of effect direction, effect size, confidence intervals, and consistency across endpoints rather than on isolated P values alone. Second, microbiota analyses are constrained by sequencing depth variability and by the fact that taxonomic shifts are not equivalent to functional shifts; the study was not designed to infer resistome or gene-level outcomes, and we intentionally avoid such claims. Third, oxidative and inflammatory biomarkers were not measured; therefore, “antioxidant mechanism” is plausible but not demonstrated. These limitations are not fatal; rather, they point to a clear next step: if the mortality interaction is repeatable, the highest-yield follow-up would be a focused perturbation–recovery experiment that integrates targeted host biomarkers with compositional-aware community analysis to test whether resilience can be predicted early (e.g., at day 20) and whether additive timing can be optimized without compromising intake.

From a production perspective, the dataset supports the view that the additive is compatible with commercial growth trajectories, whereas the timing-dependent interaction observed under enrofloxacin exposure invites a more ambitious interpretation: nutritional strategies may help stabilize outcomes under perturbation, which is the scenario where antimicrobial use decisions are often made. From a One Health perspective, that framing matters: when fluoroquinolones are under heightened stewardship scrutiny due to human-health importance, even modest improvements in robustness that reduce the need for—or the negative system-level consequences of—antimicrobial interventions can be valuable (Velazquez-Meza et al., 2022). The key remaining question is whether an optimized additive timing strategy can consistently convert subtle microbiota signatures into predictable resilience at flock scale.

## Conclusions

This study shows that an antioxidant–acidifier feed additive administered in different supplementation windows yields modest but structured production responses and timepoint-dominated cecal microbiota trajectories in broilers, with the clearest system-level signal emerging under antimicrobial perturbation. Although growth performance differences were generally small and not statistically significant in the factorial model, effect-size patterns indicated that additive timing (from week 3 onward) may exert a greater influence than supplementation maintained over the full production period. Importantly, the corrected mortality data confirmed a significant additive timing × enrofloxacin interaction, supporting the concept that nutritional strategies may contribute to robustness and recovery capacity when the gut ecosystem is challenged. Cecal microbiota profiles were driven primarily by age, and treatment-associated signatures were subtle; accordingly, the additive is best viewed as a resilience-supporting rather than microbiota-reprogramming intervention under the present conditions. Together, these findings indicate that supplementation timing is a relevant design variable when functional feed additives are evaluated under antimicrobial perturbation.

## Author contributions

Ádám Kerek contributed to the conception and design of the study, formal analysis, data curation, visualization, and preparation of the initial manuscript draft. Máté Hetyésy contributed to data acquisition and curation and critically reviewed the manuscript. Gergely Álmos Tornyos contributed to data acquisition and curation and critically reviewed the manuscript. Eszter Kaszab contributed to methodology development, provision of resources, supervision, and critical revision of the manuscript. Enikő Fehér contributed to methodology development, experimental investigation, and critical revision of the manuscript. Ákos Jerzsele contributed to study conception, provision of resources, funding acquisition, supervision, and critical revision of the manuscript. Tamás Tóth contributed to methodology development, provision of resources, supervision, and critical revision of the manuscript. Eszter Zsédely contributed to experimental investigation, provision of resources, project administration, and critical revision of the manuscript. Hedvig Fébel contributed to study conception, methodology development, supervision, and critical revision of the manuscript. All authors reviewed and explicitly approved the exact final version of the manuscript and agree to be personally accountable for all aspects of the work, including ensuring that any questions related to the accuracy or integrity of the work are appropriately investigated and resolved.

## Ethics statement

The animal experiment was approved by the competent Hungarian authority (Permit No. GY/20/02058-6/2024) and conducted in accordance with applicable national regulations and institutional animal welfare guidelines. The animals were euthanized by a trained professional using a percussive blow to the head.

## Funding sources

Project no. RRF-2.3.1-21-2022-00001 was implemented with the support provided by the Recovery and Resilience Facility (RRF), financed under the National Recovery Fund budget estimate, RRF-2.3.1-21 funding scheme.

## Disclosures

The authors declare that they have no known competing financial interests or personal relationships that could have appeared to influence the work reported in this paper.

## Data Availability

Raw sequencing reads have been deposited in the NCBI Sequence Read Archive (SRA) under BioProject accession PRJNA1393760. Processed genus-level abundance tables and analysis scripts are available from the corresponding author upon reasonable request.

## Footnotes

Supplementary material associated with this article can be found, in the online version, at doi:10.1016/j.psj.2026.107639.

## References

[bib0001] AFIA (1999).

[bib0002] Analytical Methods Committee (1991). Determination of vitamin E in animal feeding stuffs by high-performance liquid chromatography. Analyst.

[bib0003] AOAC (2012).

[bib0004] Colomer-Lluch M., Jofre J., Muniesa M. (2011). Antibiotic resistance genes in the bacteriophage DNA fraction of environmental samples. PLOS One.

[bib46] De Coster W., D’Hert S., Schultz D.T., Cruts M., Van Broeckhoven C. (2018). NanoPack: visualizing and processing long-read sequencing data. Bioinformatics.

[bib0005] Diaz Carrasco J.M., Casanova N.A., Fernández Miyakawa M.E. (2019). Microbiota, gut health and chicken productivity: what is the connection?. Microorganisms.

[bib0006] Duda-Madej A., Viscardi S., Niezgódka P., Szewczyk W., Wińska K. (2025). The impact of plant-derived polyphenols on combating efflux-mediated antibiotic resistance. Int. J. Mol. Sci..

[bib0007] Elokil A.A., Abouelezz K.F.M., Ahmad H.I., Pan Y., Li S. (2020). Investigation of the impacts of antibiotic exposure on the diversity of the gut microbiota in chicks. Animals.

[bib0008] Farkas Z., Csorba S., Vribék K., Süth M., Czudor Z., Tényi Á., Jóźwiak Á. (2025). Establishing a data infrastructure system for veterinary public health and food chain safety data through the development of a repositioning platform. Magy. Állatorvosok Lapja.

[bib0009] GBD 2021 Antimicrobial Resistance Collaborators (2024). Global burden of bacterial antimicrobial resistance 1990-2021: a systematic analysis with forecasts to 2050. Lancet.

[bib0010] Gloor G.B., Macklaim J.M., Pawlowsky-Glahn V., Egozcue J.J. (2017). Microbiome datasets are compositional: and this is not optional. Front. Microbiol..

[bib0011] Gulcin İ. (2025). Antioxidants: a comprehensive review. Arch. Toxicol..

[bib0012] Hayanti S.Y., Hidayat C., Jayanegara A., Sholikin M.M., Rusdiana S., Widyaningrum Y., Masito M., Yusriani Y., Qomariyah N., Anggraeny Y.N. (2022). Effect of vitamin E supplementation on chicken sperm quality: a meta-analysis. Vet. World.

[bib0013] He S., Ding J., Xiong Y., Liu D., Dai S., Hu H. (2020). Effects of dietary fumaric acid on growth performance, meat quality, nutrient composition and oxidative status of breast muscle in broilers under chronic heat stress. Eur. Poult. Sci..

[bib0014] Hetényi N., Bersényi A., Hullár I. (2024). Physiological effects of medium-chain fatty acids and triglycerides, and their potential use in poultry and swine nutrition: a literature review. Magy. Állatorvosok Lapja.

[bib0015] Hieu T.V., Guntoro B., Qui N.H., Quyen N.T.K., Hafiz F.A.A. (2022). The application of ascorbic acid as a therapeutic feed additive to boost immunity and antioxidant activity of poultry in heat stress environment. Vet. World.

[bib0016] Kers J.G., Velkers F.C., Fischer E.A.J., Stegeman J.A., Smidt H., Hermes G.D.A. (2022). Conserved developmental trajectories of the cecal microbiota of broiler chickens in a field study. FEMS Microbiol. Ecol..

[bib0017] Khalifa O.A., Al Wakeel R.A., Hemeda S.A., Abdel-Daim M.M., Albadrani G.M., El Askary A., Fadl S.E., Elgendey F. (2021). The impact of vitamin E and/or selenium dietary supplementation on growth parameters and expression levels of the growth-related genes in broilers. BMC Vet. Res..

[bib47] Kolmogorov M., Bickhart D.M., Behsaz B., Gurevich A., Rayko M., Shin S.B., Kuhn K., Yuan J., Polevikov E., Smith T.P.L., Pevzner P.A. (2020). metaFlye: scalable long-read metagenome assembly using repeat graphs. Nat. Meth..

[bib0018] Le Roy C.I., Woodward M.J., Ellis R.J., La Ragione R.M., Claus S.P. (2019). Antibiotic treatment triggers gut dysbiosis and modulates metabolism in a chicken model of gastro-intestinal infection. BMC Vet. Res..

[bib0019] Lőrincz E.É., Wagenhoffer Z., Zenke P. (2025). The relevance of epigenetic research in veterinary sciences : literature review. Magy. Állatorvosok Lapja.

[bib0020] Ma B., Wang D., Mei X., Lei C., Li C., Wang H. (2023). Effect of enrofloxacin on the microbiome, metabolome, and abundance of antibiotic resistance genes in the chicken Cecum. Microbiol. Spectr..

[bib0021] Mandal R.K., Jiang T., Wideman R.F., Lohrmann T., Kwon Y.M. (2020). Microbiota analysis of chickens raised under stressed conditions. Front. Vet. Sci..

[bib0022] Masenga S.K., Kabwe L.S., Chakulya M., Kirabo A. (2023). Mechanisms of oxidative stress in metabolic syndrome. Int. J. Mol. Sci..

[bib0023] Morales-Barrera E., Calhoun N., Lobato-Tapia J.L., Lucca V., Prado-Rebolledo O., Hernandez-Velasco X., Merino-Guzman R., Petrone-García V.M., Latorre J.D., Mahaffey B.D., Teague K.D., Graham L.E., Wolfenden A.D., Baxter M.F.A., Hargis B.M., Tellez G. (2016). Risks involved in the use of enrofloxacin for Salmonella Enteritidis or Salmonella Heidelberg in commercial poultry. Front. Vet. Sci..

[bib0024] Mulchandani R., Wang Y., Gilbert M., Van Boeckel T.P. (2023). Global trends in antimicrobial use in food-producing animals: 2020 to 2030. PLOS Glob. Public Health.

[bib0025] Muteeb G., Rehman M.T., Shahwan M., Aatif M. (2023). Origin of antibiotics and antibiotic resistance, and their impacts on drug development: a narrative review. Pharmaceuticals.

[bib0026] Nath S.K., Hossain M.T., Ferdous M., Siddika M.A., Hossain A., Maruf A.A., Chowdhory A.T., Nath T.C. (2023). Effects of antibiotic, acidifier, and probiotic supplementation on mortality rates, lipoprotein profile, and carcass traits of broiler chickens. Vet. Anim. Sci..

[bib0027] Oxford Nanopore Technologies (2025). Dorado. GitHub repository. https://github.com/nanoporetech/dorado.

[bib0028] Peña N., Lafuente I., Sevillano E., Feito J., Allendez G., Muñoz-Atienza E., Crispie F., Cintas L.M., Cotter P.D., Hernández P.E., Borrero J. (2025). Exploring the functional potential of the broiler gut microbiome using shotgun metagenomics. Genes.

[bib0029] Polycarpo G.V., Andretta I., Kipper M., Cruz-Polycarpo V.C., Dadalt J.C., Rodrigues P.H.M., Albuquerque R. (2017). Meta-analytic study of organic acids as an alternative performance-enhancing feed additive to antibiotics for broiler chickens. Poult. Sci..

[bib0030] Rigoulet M., Bouchez C.L., Paumard P., Ransac S., Cuvellier S., Duvezin-Caubet S., Mazat J.P., Devin A. (2020). Cell energy metabolism: an update. Biochim. Biophys. Acta Bioenerg..

[bib0031] Rostagno M.H. (2020). Effects of heat stress on the gut health of poultry. J. Anim. Sci..

[bib0032] Roth N., Käsbohrer A., Mayrhofer S., Zitz U., Hofacre C., Domig K.J. (2019). The application of antibiotics in broiler production and the resulting antibiotic resistance in Escherichia coli: a global overview. Poult. Sci..

[bib0033] Sebők C., Márton R.A., Mackei M., Neogrády Z., Mátis G. (2024). Antimicrobial peptides as new tools to combat infectious diseases. Magy. Állatorvosok Lapja.

[bib0034] Shojadoost B., Yitbarek A., Alizadeh M., Kulkarni R.R., Astill J., Boodhoo N., Sharif S. (2021). Centennial review: effects of vitamins A, D, E, and C on the chicken immune system. Poult. Sci..

[bib0035] Skinner J.T., Izat A.L., Waldroup P.W. (1991). Research note: fumaric acid enhances performance of broiler chickens. Poult. Sci..

[bib0036] Somogyi F., Farkas O. (2025). Possibilities of in vitro modelling of porcine infectious enteropathies : literature review. Magy. Állatorvosok Lapja.

[bib0037] Subhadra B., Oh M.H., Choi C.H. (2019). RND efflux pump systems in acinetobacter, with special emphasis on their role in quorum sensing. J. Bacteriol. Virol..

[bib0038] Tang K.W.K., Millar B.C., Moore J.E. (2023). Antimicrobial resistance (AMR). Br. J. Biomed. Sci..

[bib0039] Temmerman R., Ghanbari M., Antonissen G., Schatzmayr G., Duchateau L., Haesebrouck F., Garmyn A., Devreese M. (2022). Dose-dependent impact of enrofloxacin on broiler chicken gut resistome is mitigated by synbiotic application. Front. Microbiol..

[bib0040] Varga-Balogh O., Olasz F. (2025). Role of microbiota in cattle reproduction : literature review. Magy. Állatorvosok Lapja.

[bib0041] Velazquez-Meza M.E., Galarde-López M., Carrillo-Quiróz B., Alpuche-Aranda C.M. (2022). Antimicrobial resistance: one health approach. Vet. World.

[bib0042] Vraka C., Nics L., Wagner K.-H., Hacker M., Wadsak W., Mitterhauser M. (2017). LogP, a yesterday’s value?. Nucl. Med. Biol..

[bib0043] Xiao Y., Gao X., Yuan J. (2024). Comparative study of an antioxidant compound and ethoxyquin on feed oxidative stability and on performance, antioxidant capacity, and intestinal health in starter broiler chickens. Antioxidants.

[bib0044] Yang H., Leng J., Liu N., Huang L. (2024). Editorial: free radicals and antioxidants in diseases associated with immune dysfunction, inflammatory process, and aberrant metabolism. Front. Endocrinol..

[bib0045] Zhou J., Li Z., Guo W., Wang Y., Liu R., Huang X., Li Y., Yang X., Liu L., Liu Y., Xu X. (2024). Nano vitamin E improved the antioxidant capacity of broiler chickens. J. Anim. Sci..

