# Supplementary material for: Timing of an antioxidant–acidifier feed additive modulates growth performance and cecal microbiota in broiler chickens during enrofloxacin exposure
Source: Poult Sci. 2026 Aug 17;105(11):107639. doi: 10.1016/j.psj.2026.107639 (PMC13590082; doi:10.1016/j.psj.2026.107639)
Supplement: Supplementary file 1 [file mmc3.docx]

**Supplementary Table S1.** Overview of experimental design and sampling scheme.

| **Treatment** | **Treatment description** | **Feed additive window** | **Enrofloxacin window** | **Pens**  **(n)** | **Birds/pen (n)** | **Planned cecal samples/time point (n)** |
| --- | --- | --- | --- | --- | --- | --- |
| Control (C) | Basal diet; no feed additive; no enrofloxacin | None | None | 4 | 50 | 8 |
| Enrofloxacin (EF) | Basal diet; enrofloxacin via drinking water | None | d15 to d19 | 4 | 50 | 8 |
| Feed additive  4 week (A4) | Basal diet + antioxidant–acidifier additive | d15 to d42 | None | 4 | 50 | 8 |
| A4 + EF | Basal diet + additive; enrofloxacin via drinking water | d15 to d42 | d15 to d19 | 4 | 50 | 8 |
| Feed additive  6 week (A6) | Basal diet + antioxidant–acidifier additive | d1 to d42 | None | 4 | 50 | 8 |
| A6 + EF | Basal diet + additive; enrofloxacin via drinking water | d1 to d42 | d15 to d19 | 4 | 50 | 8 |

Notes: 6 treatments; 4 replicate pens/treatment; 50 birds/pen (total 1,200). Cecal sampling at d14, d20, and d42 from 2 birds/pen/time point (planned: 48 samples/time point; 144 total). Pen-level microbiota profiles were computed per time point as one aggregated genus profile per pen (24 profiles/time point; 72 total).

**Supplementary Table S2**. Sample-level read counts, taxonomic classification output (Kraken2/Bracken) and metaQUAST assembly statistics for cecal metagenome assemblies. Counts are reported per sequenced cecal sample. “Unclassified/Unmapped” corresponds to reads not assigned at the genus rank by the taxonomic classification workflow. “Classified” = Total reads − Unclassified/Unmapped. Assembly summary and reference-based quality metrics are reported for each de novo metagenome assembly generated from cecal shotgun metagenomic reads. Metrics were extracted from the metaQUAST report (minimum contig length threshold = 500 bp) and include basic assembly statistics (e.g., number of contigs, total assembly length, largest contig, N50/L50), alignment-derived measures (e.g., genome fraction, duplication ratio, total aligned length), error and structural indicators (e.g., misassembles, mismatches and indels per 100 kbp), and unaligned sequence statistics. Sample identifiers and associated experimental metadata (sampling day and treatment labels) are provided to facilitate cross-referencing with diversity and compositional analyses.

| **sample** | **timepoint** | **Treatment** | **pen** | **Total reads** | **Classified reads** | **Classified (%)** | **Unclassified/Unmapped reads** | **Unclassified/Unmapped (%)** | **contigs** | **Total length** | **N50** | **L50** | **Genome fraction (%)** |
| --- | --- | --- | --- | --- | --- | --- | --- | --- | --- | --- | --- | --- | --- |
| I_1 | d14 | Control (C) | 1 | 90 022 | 78 367 | 87.05 | 11 655 | 12.95 | 819 | 20591402 | 46380 | 60 | 8.843 |
| I_2 | d14 | Control (C) | 1 | 48 903 | 38 793 | 79.33 | 10 110 | 20.67 | 245 | 3527746 | 22083 | 41 | 3.683 |
| I_13 | d14 | Control (C) | 7 | 82 049 | 61 519 | 74.98 | 20 530 | 25.02 | 53 | 5692418 | 506611 | 3 | 8.366 |
| I_14 | d14 | Control (C) | 7 | 189 689 | 86 850 | 45.79 | 102 839 | 54.21 | 339 | 6529043 | 97717 | 20 | 9.013 |
| I_25 | d14 | Control (C) | 13 | 114 092 | 80 569 | 70.62 | 33 523 | 29.38 | 182 | 1429652 | 10755 | 45 | 1.436 |
| I_37 | d14 | Control (C) | 19 | 102 559 | 51 386 | 50.10 | 51 173 | 49.90 | 1353 | 18132936 | 20105 | 159 | 8.219 |
| I_38 | d14 | Control (C) | 19 | 433 408 | 110 925 | 25.59 | 322 483 | 74.41 | 1798 | 11285973 | 8130 | 439 | 0.818 |
| I_3 | d14 | Enrofloxacin (EF) | 2 | 195 956 | 97 067 | 49.54 | 98 889 | 50.46 | 623 | 8291196 | 27267 | 90 | 6.257 |
| I_4 | d14 | Enrofloxacin (EF) | 2 | 25 212 | 12 913 | 51.22 | 12 299 | 48.78 | 44 | 371602 | 12267 | 11 | 0.396 |
| I_15 | d14 | Enrofloxacin (EF) | 8 | 312 186 | 83 324 | 26.69 | 228 862 | 73.31 | 932 | 7023257 | 10471 | 183 | 0.885 |
| I_16 | d14 | Enrofloxacin (EF) | 8 | 40 680 | 23 055 | 56.67 | 17 625 | 43.33 | 161 | 4404493 | 651728 | 2 | 4.018 |
| I_27 | d14 | Enrofloxacin (EF) | 14 | 180 080 | 79 259 | 44.01 | 100 821 | 55.99 | 441 | 5652463 | 20369 | 74 | 6.151 |
| I_28 | d14 | Enrofloxacin (EF) | 14 | 111 776 | 65 182 | 58.31 | 46 594 | 41.69 | 814 | 14466760 | 44755 | 30 | 8.338 |
| I_39 | d14 | Enrofloxacin (EF) | 20 | 322 260 | 114 412 | 35.50 | 207 848 | 64.50 | 1152 | 7171284 | 7835 | 262 | 1.566 |
| I_40 | d14 | Enrofloxacin (EF) | 20 | 117 803 | 48 231 | 40.94 | 69 572 | 59.06 | 493 | 5320545 | 17505 | 86 | 5.64 |
| I_9 | d14 | A4 | 5 | 251 584 | 57 421 | 22.82 | 194 163 | 77.18 | 503 | 2559217 | 6374 | 122 | 0.265 |
| I_10 | d14 | A4 | 5 | 88 669 | 32 421 | 36.56 | 56 248 | 63.44 | 379 | 4246335 | 16373 | 74 | 3.342 |
| I_21 | d14 | A4 | 11 | 58 588 | 35 390 | 60.40 | 23 198 | 39.60 | 287 | 9208546 | 97361 | 28 | 11.735 |
| I_22 | d14 | A4 | 11 | 77 570 | 32 658 | 42.10 | 44 912 | 57.90 | 1559 | 8852031 | 6916 | 379 | 3.29 |
| I_33 | d14 | A4 | 17 | 319 856 | 125 463 | 39.22 | 194 393 | 60.78 | 2026 | 15980564 | 10940 | 413 | 7.396 |
| I_34 | d14 | A4 | 17 | 241 530 | 158 022 | 65.43 | 83 508 | 34.57 | 809 | 6544979 | 11672 | 161 | 5.228 |
| I_45 | d14 | A4 | 23 | 440 592 | 267 222 | 60.65 | 173 370 | 39.35 | 4202 | 36448237 | 12510 | 517 | 9.909 |
| I_46 | d14 | A4 | 23 | 208 389 | 81 605 | 39.16 | 126 784 | 60.84 | 1589 | 19772142 | 22814 | 177 | 9.641 |
| I_5 | d14 | A4+EF | 3 | 126 235 | 104 963 | 83.15 | 21 272 | 16.85 | 627 | 10825558 | 67238 | 5 | 8.679 |
| I_6 | d14 | A4+EF | 3 | 64 464 | 35 537 | 55.13 | 28 927 | 44.87 | 273 | 6937993 | 156120 | 9 | 9.351 |
| I_17 | d14 | A4+EF | 9 | 230 762 | 96 650 | 41.88 | 134 112 | 58.12 | 565 | 6443286 | 20899 | 82 | 6.971 |
| I_18 | d14 | A4+EF | 9 | 106 811 | 58 504 | 54.77 | 48 307 | 45.23 | 779 | 8785953 | 20093 | 113 | 7.905 |
| I_29 | d14 | A4+EF | 15 | 282 471 | 127 719 | 45.21 | 154 752 | 54.79 | 1582 | 12850104 | 11691 | 253 | 9.194 |
| I_30 | d14 | A4+EF | 15 | 100 086 | 66 252 | 66.20 | 33 834 | 33.80 | 537 | 10740244 | 68185 | 2 | 8.566 |
| I_41 | d14 | A4+EF | 21 | 339 502 | 113 609 | 33.46 | 225 893 | 66.54 | 1245 | 9119823 | 9924 | 239 | 6.191 |
| I_42 | d14 | A4+EF | 21 | 52 122 | 21 986 | 42.18 | 30 136 | 57.82 | 239 | 1310707 | 6461 | 60 | 0.601 |
| I_11 | d14 | A6 | 6 | 103 548 | 56 152 | 54.23 | 47 396 | 45.77 | 787 | 11800800 | 31765 | 89 | 7.776 |
| I_12 | d14 | A6 | 6 | 128 346 | 57 815 | 45.05 | 70 531 | 54.95 | 1318 | 19695321 | 30461 | 147 | 8.585 |
| I_23 | d14 | A6 | 12 | 155 766 | 40 524 | 26.02 | 115 242 | 73.98 | 288 | 1351369 | 4979 | 77 | 0.236 |
| I_24 | d14 | A6 | 12 | 241 936 | 70 530 | 29.15 | 171 406 | 70.85 | 1274 | 10862516 | 13031 | 182 | 1.943 |
| I_35 | d14 | A6 | 18 | 323 628 | 223 702 | 69.12 | 99 926 | 30.88 | 782 | 7966148 | 20917 | 93 | 7.077 |
| I_36 | d14 | A6 | 18 | 323 930 | 135 246 | 41.75 | 188 684 | 58.25 | 1591 | 12795052 | 11154 | 267 | 7.034 |
| I_47 | d14 | A6 | 24 | 114 459 | 80 291 | 70.15 | 34 168 | 29.85 | 1359 | 16415576 | 22509 | 159 | 9.418 |
| I_48 | d14 | A6 | 24 | 288 186 | 123 908 | 43.00 | 164 278 | 57.00 | 3716 | 33773050 | 13697 | 588 | 9.605 |
| I_7 | d14 | A6+EF | 4 | 78 971 | 41 948 | 53.12 | 37 023 | 46.88 | 515 | 8339048 | 33467 | 23 | 4.266 |
| I_19 | d14 | A6+EF | 10 | 110 307 | 61 817 | 56.04 | 48 490 | 43.96 | 1100 | 18800400 | 46449 | 46 | 10.396 |
| I_20 | d14 | A6+EF | 10 | 212 658 | 114 455 | 53.82 | 98 203 | 46.18 | 429 | 5326072 | 21808 | 67 | 6.126 |
| I_31 | d14 | A6+EF | 16 | 343 440 | 195 848 | 57.03 | 147 592 | 42.97 | 1073 | 13993286 | 30998 | 68 | 13.152 |
| I_32 | d14 | A6+EF | 16 | 241 829 | 71 661 | 29.63 | 170 168 | 70.37 | 789 | 4206945 | 6513 | 195 | 1.186 |
| I_43 | d14 | A6+EF | 22 | 330 873 | 243 910 | 73.72 | 86 963 | 26.28 | 3353 | 23661917 | 9507 | 653 | 6.947 |
| I_44 | d14 | A6+EF | 22 | 89 553 | 49 846 | 55.66 | 39 707 | 44.34 | 660 | 8365474 | 27898 | 84 | 7.222 |
| II_1 | d20 | Control (C) | 1 | 169 583 | 86 955 | 51.28 | 82 628 | 48.72 | 2059 | 34083144 | 33399 | 162 | 14.403 |
| II_2 | d20 | Control (C) | 1 | 376 833 | 202 694 | 53.79 | 174 139 | 46.21 | 4419 | 47681649 | 17379 | 438 | 17.733 |
| II_13 | d20 | Control (C) | 7 | 459 946 | 252 094 | 54.81 | 207 852 | 45.19 | 5520 | 49968159 | 13776 | 574 | 11.876 |
| II_14 | d20 | Control (C) | 7 | 477 625 | 241 423 | 50.55 | 236 202 | 49.45 | 5088 | 53968746 | 16493 | 496 | 11.166 |
| II_25 | d20 | Control (C) | 13 | 197 763 | 92 548 | 46.80 | 105 215 | 53.20 | 1878 | 20886630 | 18587 | 214 | 6.697 |
| II_26 | d20 | Control (C) | 13 | 96 625 | 36 966 | 38.26 | 59 659 | 61.74 | 197 | 4657819 | 837408 | 2 | 0.473 |
| II_37 | d20 | Control (C) | 19 | 151 562 | 88 234 | 58.22 | 63 328 | 41.78 | 1954 | 22924803 | 18253 | 222 | 13.321 |
| II_38 | d20 | Control (C) | 19 | 56 515 | 27 688 | 48.99 | 28 827 | 51.01 | 208 | 3389602 | 33699 | 29 | 3.509 |
| II_3 | d20 | Enrofloxacin (EF) | 2 | 99 920 | 41 048 | 41.08 | 58 872 | 58.92 | 1076 | 14993409 | 23025 | 151 | 4.88 |
| II_4 | d20 | Enrofloxacin (EF) | 2 | 139 592 | 64 323 | 46.08 | 75 269 | 53.92 | 1490 | 16027270 | 14743 | 164 | 2.5 |
| II_15 | d20 | Enrofloxacin (EF) | 8 | 122 125 | 42 433 | 34.75 | 79 692 | 65.25 | 1082 | 10365049 | 14744 | 181 | 6.961 |
| II_16 | d20 | Enrofloxacin (EF) | 8 | 336 280 | 239 656 | 71.27 | 96 624 | 28.73 | 4118 | 41752111 | 16646 | 390 | 14.495 |
| II_27 | d20 | Enrofloxacin (EF) | 14 | 338 866 | 167 454 | 49.42 | 171 412 | 50.58 | 3977 | 48292175 | 21844 | 396 | 11.738 |
| II_28 | d20 | Enrofloxacin (EF) | 14 | 339 692 | 208 784 | 61.46 | 130 908 | 38.54 | 3936 | 49591668 | 21309 | 356 | 10.739 |
| II_39 | d20 | Enrofloxacin (EF) | 20 | 44 107 | 14 904 | 33.79 | 29 203 | 66.21 | 120 | 573182 | 5955 | 31 | 0.206 |
| II_40 | d20 | Enrofloxacin (EF) | 20 | 108 686 | 43 880 | 40.37 | 64 806 | 59.63 | 697 | 8189333 | 21141 | 56 | 4.972 |
| II_9 | d20 | A4 | 5 | 493 857 | 200 688 | 40.64 | 293 169 | 59.36 | 6285 | 66942157 | 17018 | 536 | 15.484 |
| II_10 | d20 | A4 | 5 | 148 035 | 69 661 | 47.06 | 78 374 | 52.94 | 1570 | 25694363 | 57917 | 26 | 13.833 |
| II_21 | d20 | A4 | 11 | 229 238 | 133 273 | 58.14 | 95 965 | 41.86 | 1408 | 20245696 | 28956 | 83 | 13.972 |
| II_22 | d20 | A4 | 11 | 319 334 | 184 694 | 57.84 | 134 640 | 42.16 | 3543 | 42072257 | 20888 | 284 | 14.963 |
| II_33 | d20 | A4 | 17 | 47 029 | 37 345 | 79.41 | 9 684 | 20.59 | 375 | 4445920 | 21241 | 51 | 3.441 |
| II_34 | d20 | A4 | 17 | 50 787 | 23 394 | 46.06 | 27 393 | 53.94 | 223 | 1780105 | 11064 | 48 | 1.297 |
| II_45 | d20 | A4 | 23 | 81 256 | 33 191 | 40.85 | 48 065 | 59.15 | 898 | 8418067 | 16316 | 119 | 8.105 |
| II_46 | d20 | A4 | 23 | 91 531 | 48 471 | 52.96 | 43 060 | 47.04 | 385 | 6173221 | 43290 | 38 | 8.255 |
| II_5 | d20 | A4+EF | 3 | 47 487 | 24 808 | 52.24 | 22 679 | 47.76 | 427 | 9582478 | 42272 | 43 | 5.062 |
| II_6 | d20 | A4+EF | 3 | 325 137 | 154 705 | 47.58 | 170 432 | 52.42 | 2818 | 25507669 | 12447 | 455 | 9.009 |
| II_17 | d20 | A4+EF | 9 | 286 074 | 145 176 | 50.75 | 140 898 | 49.25 | 2724 | 28460646 | 15406 | 426 | 6.091 |
| II_18 | d20 | A4+EF | 9 | 175 050 | 86 601 | 49.47 | 88 449 | 50.53 | 1408 | 25111589 | 39370 | 115 | 13.483 |
| II_29 | d20 | A4+EF | 15 | 266 281 | 165 299 | 62.08 | 100 982 | 37.92 | 1468 | 18545667 | 27587 | 48 | 9.724 |
| II_30 | d20 | A4+EF | 15 | 177 062 | 107 204 | 60.55 | 69 858 | 39.45 | 1241 | 14678212 | 22223 | 52 | 9.717 |
| II_41 | d20 | A4+EF | 21 | 57 420 | 30 212 | 52.62 | 27 208 | 47.38 | 649 | 6948788 | 16575 | 111 | 4.085 |
| II_42 | d20 | A4+EF | 21 | 55 711 | 21 489 | 38.57 | 34 222 | 61.43 | 214 | 1381837 | 8409 | 46 | 0.258 |
| II_11 | d20 | A6 | 6 | 148 687 | 70 502 | 47.42 | 78 185 | 52.58 | 772 | 8682708 | 17935 | 75 | 2.637 |
| II_12 | d20 | A6 | 6 | 154 340 | 77 806 | 50.41 | 76 534 | 49.59 | 2397 | 24306579 | 15398 | 335 | 3.915 |
| II_23 | d20 | A6 | 12 | 319 386 | 214 412 | 67.13 | 104 974 | 32.87 | 5722 | 61446183 | 16157 | 825 | 14.426 |
| II_24 | d20 | A6 | 12 | 286 905 | 201 182 | 70.12 | 85 723 | 29.88 | 3279 | 37757698 | 20013 | 307 | 14.352 |
| II_35 | d20 | A6 | 18 | 40 606 | 27 915 | 68.75 | 12 691 | 31.25 | 294 | 3756239 | 20265 | 55 | 1.748 |
| II_36 | d20 | A6 | 18 | 22 876 | 7 757 | 33.91 | 15 119 | 66.09 | 28 | 295194 | 20401 | 6 | 0.034 |
| II_47 | d20 | A6 | 24 | 61 639 | 43 935 | 71.28 | 17 704 | 28.72 | 646 | 6291461 | 15195 | 112 | 4.034 |
| II_48 | d20 | A6 | 24 | 65 801 | 32 728 | 49.74 | 33 073 | 50.26 | 782 | 6562758 | 12515 | 137 | 3.651 |
| II_7 | d20 | A6+EF | 4 | 259 302 | 120 392 | 46.43 | 138 910 | 53.57 | 2100 | 22086983 | 16881 | 268 | 7.668 |
| II_8 | d20 | A6+EF | 4 | 309 458 | 205 239 | 66.32 | 104 219 | 33.68 | 2082 | 22966400 | 18341 | 231 | 13.092 |
| II_19 | d20 | A6+EF | 10 | 292 085 | 144 858 | 49.59 | 147 227 | 50.41 | 2282 | 25706621 | 19642 | 237 | 8.916 |
| II_20 | d20 | A6+EF | 10 | 113 480 | 51 923 | 45.76 | 61 557 | 54.24 | 993 | 11284529 | 17221 | 93 | 5.435 |
| II_31 | d20 | A6+EF | 16 | 155 549 | 109 387 | 70.32 | 46 162 | 29.68 | 2478 | 27759989 | 17408 | 289 | 10.55 |
| II_32 | d20 | A6+EF | 16 | 91 428 | 59 866 | 65.48 | 31 562 | 34.52 | 541 | 6285724 | 18190 | 91 | 7.552 |
| II_43 | d20 | A6+EF | 22 | 66 320 | 32 694 | 49.30 | 33 626 | 50.70 | 569 | 7211904 | 19697 | 99 | 4.885 |
| II_44 | d20 | A6+EF | 22 | 46 657 | 22 017 | 47.19 | 24 640 | 52.81 | 262 | 2976201 | 18009 | 49 | 3.47 |
| III_1 | d42 | Control (C) | 1 | 53 276 | 14 644 | 27.49 | 38 632 | 72.51 | 540 | 3899713 | 9736 | 119 | 0.843 |
| III_2 | d42 | Control (C) | 1 | 85 956 | 25 256 | 29.38 | 60 700 | 70.62 | 1311 | 11086289 | 13279 | 247 | 2.069 |
| III_13 | d42 | Control (C) | 7 | 213 910 | 147 728 | 69.06 | 66 182 | 30.94 | 2611 | 30010581 | 18084 | 384 | 6.377 |
| III_14 | d42 | Control (C) | 7 | 287 057 | 99 156 | 34.54 | 187 901 | 65.46 | 2989 | 30574843 | 17588 | 352 | 4.464 |
| III_25 | d42 | Control (C) | 13 | 405 631 | 137 940 | 34.01 | 267 691 | 65.99 | 5545 | 58840191 | 18450 | 623 | 13.535 |
| III_26 | d42 | Control (C) | 13 | 275 181 | 109 551 | 39.81 | 165 630 | 60.19 | 2436 | 39882943 | 32198 | 155 | 11.615 |
| III_37 | d42 | Control (C) | 19 | 123 820 | 42 404 | 34.25 | 81 416 | 65.75 | 1786 | 18525598 | 14738 | 332 | 2.804 |
| III_38 | d42 | Control (C) | 19 | 281 097 | 183 520 | 65.29 | 97 577 | 34.71 | 2989 | 41377825 | 28563 | 275 | 2.898 |
| III_3 | d42 | Enrofloxacin (EF) | 2 | 120 495 | 29 423 | 24.42 | 91 072 | 75.58 | 1620 | 12716790 | 10946 | 263 | 1.582 |
| III_4 | d42 | Enrofloxacin (EF) | 2 | 92 355 | 42 075 | 45.56 | 50 280 | 54.44 | 1891 | 17161532 | 13268 | 287 | 1.863 |
| III_15 | d42 | Enrofloxacin (EF) | 8 | 108 954 | 38 956 | 35.75 | 69 998 | 64.25 | 1762 | 17882228 | 14834 | 320 | 3.67 |
| III_16 | d42 | Enrofloxacin (EF) | 8 | 197 891 | 74 282 | 37.54 | 123 609 | 62.46 | 1983 | 18697778 | 13845 | 376 | 3.943 |
| III_27 | d42 | Enrofloxacin (EF) | 14 | 107 622 | 47 797 | 44.41 | 59 825 | 55.59 | 1489 | 16164001 | 14886 | 221 | 1.876 |
| III_28 | d42 | Enrofloxacin (EF) | 14 | 130 277 | 82 791 | 63.55 | 47 486 | 36.45 | 1556 | 16961967 | 17749 | 177 | 2.598 |
| III_39 | d42 | Enrofloxacin (EF) | 20 | 205 887 | 139 384 | 67.70 | 66 503 | 32.30 | 2175 | 24943526 | 19208 | 213 | 6.859 |
| III_40 | d42 | Enrofloxacin (EF) | 20 | 399 875 | 194 929 | 48.75 | 204 946 | 51.25 | 3649 | 38289715 | 16753 | 398 | 10.781 |
| III_9 | d42 | A4 | 5 | 79 749 | 24 829 | 31.13 | 54 920 | 68.87 | 1095 | 11850787 | 16034 | 168 | 1.967 |
| III_10 | d42 | A4 | 5 | 19 461 | 7 345 | 37.74 | 12 116 | 62.26 | 37 | 285543 | 10950 | 6 | 0.03 |
| III_21 | d42 | A4 | 11 | 273 956 | 80 546 | 29.40 | 193 410 | 70.60 | 1566 | 11602010 | 10771 | 244 | 1.522 |
| III_22 | d42 | A4 | 11 | 314 026 | 96 500 | 30.73 | 217 526 | 69.27 | 3770 | 37056074 | 14595 | 501 | 4.429 |
| III_33 | d42 | A4 | 17 | 349 827 | 151 592 | 43.33 | 198 235 | 56.67 | 1918 | 23225891 | 22013 | 173 | 4.185 |
| III_34 | d42 | A4 | 17 | 329 938 | 94 800 | 28.73 | 235 138 | 71.27 | 4609 | 43983652 | 14657 | 538 | 3.213 |
| III_45 | d42 | A4 | 23 | 178 617 | 75 327 | 42.17 | 103 290 | 57.83 | 2611 | 26138792 | 15300 | 358 | 2.597 |
| III_46 | d42 | A4 | 23 | 286 390 | 84 077 | 29.36 | 202 313 | 70.64 | 2932 | 27079023 | 14231 | 359 | 5.17 |
| III_5 | d42 | A4+EF | 3 | 32 224 | 19 288 | 59.86 | 12 936 | 40.14 | 334 | 2474005 | 9587 | 77 | 0.27 |
| III_6 | d42 | A4+EF | 3 | 4 020 | 1 682 | 41.84 | 2 338 | 58.16 | 3 | 16024 | 4431 | 2 | 0.01 |
| III_17 | d42 | A4+EF | 9 | 126 473 | 47 133 | 37.27 | 79 340 | 62.73 | 1022 | 13845145 | 22396 | 116 | 2.042 |
| III_18 | d42 | A4+EF | 9 | 304 030 | 129 300 | 42.53 | 174 730 | 57.47 | 2588 | 28279206 | 16938 | 275 | 4.461 |
| III_29 | d42 | A4+EF | 15 | 115 254 | 70 765 | 61.40 | 44 489 | 38.60 | 1216 | 16820618 | 25210 | 119 | 3.594 |
| III_30 | d42 | A4+EF | 15 | 251 107 | 144 470 | 57.53 | 106 637 | 42.47 | 2069 | 23331018 | 23964 | 186 | 3.58 |
| III_41 | d42 | A4+EF | 21 | 312 189 | 118 803 | 38.05 | 193 386 | 61.95 | 2211 | 27289891 | 21296 | 258 | 5.233 |
| III_42 | d42 | A4+EF | 21 | 150 853 | 62 425 | 41.38 | 88 428 | 58.62 | 1508 | 22127626 | 26495 | 98 | 4.341 |
| III_11 | d42 | A6 | 6 | 35 483 | 8 695 | 24.50 | 26 788 | 75.50 | 349 | 1763444 | 6144 | 91 | 0.03 |
| III_12 | d42 | A6 | 6 | 50 361 | 16 078 | 31.93 | 34 283 | 68.07 | 346 | 2230359 | 8273 | 77 | 0.759 |
| III_23 | d42 | A6 | 12 | 336 365 | 142 806 | 42.46 | 193 559 | 57.54 | 1949 | 25269589 | 26526 | 202 | 3.692 |
| III_24 | d42 | A6 | 12 | 231 901 | 94 281 | 40.66 | 137 620 | 59.34 | 2206 | 22989439 | 16678 | 308 | 3.663 |
| III_35 | d42 | A6 | 18 | 268 309 | 142 756 | 53.21 | 125 553 | 46.79 | 2946 | 30045779 | 14947 | 353 | 4.185 |
| III_36 | d42 | A6 | 18 | 125 479 | 57 396 | 45.74 | 68 083 | 54.26 | 1632 | 14632821 | 12267 | 280 | 1.835 |
| III_47 | d42 | A6 | 24 | 98 885 | 32 884 | 33.25 | 66 001 | 66.75 | 505 | 4552173 | 13298 | 97 | 1.426 |
| III_48 | d42 | A6 | 24 | 145 884 | 72 657 | 49.80 | 73 227 | 50.20 | 762 | 13096575 | 44526 | 30 | 4.142 |
| III_7 | d42 | A6+EF | 4 | 3 621 | 1 123 | 31.01 | 2 498 | 68.99 | 6 | 23175 | 4010 | 3 | 0.02 |
| III_8 | d42 | A6+EF | 4 | 44 783 | 11 501 | 25.68 | 33 282 | 74.32 | 353 | 1825487 | 6327 | 90 | 0.203 |
| III_19 | d42 | A6+EF | 10 | 131 725 | 52 285 | 39.69 | 79 440 | 60.31 | 758 | 15229881 | 41857 | 62 | 4.173 |
| III_20 | d42 | A6+EF | 10 | 109 742 | 46 844 | 42.69 | 62 898 | 57.31 | 844 | 21615796 | 141422 | 26 | 3.38 |
| III_31 | d42 | A6+EF | 16 | 299 292 | 114 482 | 38.25 | 184 810 | 61.75 | 3205 | 32001798 | 17454 | 303 | 4.318 |
| III_32 | d42 | A6+EF | 16 | 374 735 | 157 142 | 41.93 | 217 593 | 58.07 | 3736 | 37375987 | 15426 | 450 | 7.797 |
| III_43 | d42 | A6+EF | 22 | 162 042 | 64 246 | 39.65 | 97 796 | 60.35 | 1882 | 19252889 | 16023 | 294 | 2.311 |
| III_44 | d42 | A6+EF | 22 | 46 795 | 21 809 | 46.61 | 24 986 | 53.39 | 561 | 9714344 | 27274 | 81 | 1.805 |

**Supplementary Figure S1.** Sequencing depth across sampling timepoints (genus level). Boxplots show the distribution of genus-classified reads per sample at d14, d20, and d42. The center line indicates the median, box limits denote the interquartile range, and whiskers represent the range excluding outliers (not shown).

**Supplementary Figure S2.** Shannon alpha diversity (genus level) by treatment group at (A) d14, (B) d20, and (C) d42. Shannon index was calculated from pen-level genus profiles (two birds aggregated per pen and time point). Boxes show median and interquartile range; whiskers indicate 1.5×IQR; points represent individual pens (*n*=4 pens/treatment).
